# Constructing Schwartz values framework using the Rokeach values survey: Human value measurement in the longitudinal internet survey for social sciences

**DOI:** 10.1371/journal.pone.0329179

**Published:** 2025-08-12

**Authors:** Oscar Smallenbroek, Ingmar Leijen, A. Stanciu, Hester van Herk, A. Bardi

**Affiliations:** 1 Joint Research Centre, European Commission, Ispra, Italy; 2 School of Business and Economics, Vrije Universiteit Amsterdam, Amsterdam, Netherlands; 3 Faculty of Humanities, Education and Social Sciences (FHSE), University of Luxembourg, Esch-sur-Alzette, Luxembourg; 4 Department of Psychology, Royal Holloway University of London, Egham, England; Hunan University, CHINA

## Abstract

This paper assesses and validates indexes of human values as conceptualized in the Schwartz framework using the Rokeach Value Survey in the publicly available LISS panel data (www.lisspanel.nl), thereby opening an avenue for researchers to relate values to a wide range of attitudes, traits, and behaviors with the most widely used and tested values theory. We start with a theoretical screening of the value items, after which we use multi-dimensional scaling to assess the item’s correlation structure. We developed reliable and valid indexes for 8 of the 10 Schwartz values, of which universalism, achievement, benevolence, and conformity contain 3 or more items, and self-direction, stimulation, hedonism, and security contain 1 or 2 items. We assess the RVS-based indexes by comparing them with other Schwartz indexes in the World Value Survey and the European Social Survey data. Using regressions, all 8 values also show the expected relationships with demographics, political orientation, and personality traits. By tailoring the RVS to align with the Schwartz value structure, researchers can use a well-established framework for assessing human values longitudinally and cross-sectionally, enabling better comparisons and insights across studies and time periods.

## Introduction

The Schwartz [1] values framework is currently the most widely accepted and extensively studied theory and measurement of human values [2], leading researchers to favour it’s measurement instruments over earlier instruments—even though the latter could still yield valuable insights, particularly given the large amount of existing data. One such measure is the Rokeach Values Survey [3] which has been used for since 2008 in the Longitudinal Internet Survey for Social Sciences [4] – an ongoing longitudinal survey based on a representative Dutch sample. This paper establishes indexes of the Schwartz [1] values with items from the Rokeach Value Survey, thereby enabling use of this source within the widely validated conceptualizations of the Schwartz values theory [1].

Establishing a meaningful link between the RVS items and the Schwartz values (Table 1) can provide many researchers a rich panel dataset containing over 10,000 respondents aged 16 + covering 2008 to the present in the Netherlands. The LISS is a high-quality open source, nationally representative panel covering personality, political behavior and attitudes as well as a range of economic and demographic topics. It includes high-quality documentation in English. Hence, it provides a great source to study how and when values change as well as value’s relationship with emotions, personality traits, subjective well-being, political affiliation and voting choice. The LISS also includes many one-off questionnaires requested by researchers which add to this rich panel data and address current issues such as attitudes on gender equality, climate change, the green energy transition, population aging, nationalism, populism and democracy, immigration and others. In fact, the LISS data covers most topics in the values literature, as identified by Schwartz and Sagiv [1].

**Table 1:** Definitions of the Schwartz value types in the revised theory.

Value	Definition
Self-direction	Independent thought and action, choosing, creating, and exploring
Stimulation	Excitement, novelty, and challenge in life
Hedonism	Pleasure and sensuous gratification for oneself
Achievement	Personal success through demonstrating competence according to social standards
Power	Social status and prestige, control or dominance over people and resources
Security	Safety, harmony, and stability of society, relationships, and self
Tradition	Respect of, commitment to, and acceptance of the customs and ideas that traditional culture or religion provides
Conformity	Restraint of actions, inclinations, and impulses likely to upset or harm others or violate social expectations or norms
Benevolence	Preservation and enhancement of the welfare of people with whom one is in frequent personal contact
Universalism	Understanding, appreciation, tolerance, and protection for the welfare of all people and of nature

Note: Adapted from Sagiv and Schwartz (2022).

The Rokeach Values Survey [3] is still in use today (e.g., [5–9]). It is widely used for assessing human values in work related assessments (e.g., [10]), marketing and advertising (e.g., [11]), social psychology (reviewed in [12]), and cross-cultural research (e.g., [13]). However, it has long been considered an outdated instrument since Schwartz and Bilsky [14,15] developed a theory of the contents and structure of values using a multinational sample and created a well validated framework that has been widely adopted [16,17]. These seminal papers established the Schwartz values framework as the state-of-the-art, spurring on the development of several instruments, initially heavily drawing on the RVS to eventually changing many items, their presentation and scales (e.g., [18–22]). Nevertheless, the historical continuity between the measurements suggests potential for a successful mapping of RVS items onto the Schwartz framework. Hence, in this paper, we investigate which items of the RVS in the LISS panel best represent the Schwartz values structure to help researchers use one agreed way of allocating the RVS items in the LISS to the Schwartz [23]values.

### The evolution of the theory and measurement of human values from Rokeach to Schwartz

The Rokeach Values Survey was developed in the 1970’s by the social psychologist Milton Rokeach [3] building on previous work by [24] and [25].The RVS differentiates between 18 terminal values (desired end-states of existence – the ultimate goals that individuals aspire to achieve), and 18 instrumental values (preferable modes of behavior that are means to achieve the terminal goals) which participants rank as separate lists. Theoretically, a further distinction was made between personal goals and social goals. Some of the items used in the RVS have been criticized, as they are emotional states (e.g., ‘happiness’, ‘inner harmony’) while values are defined as broad goals that guide people’s lives [16].

**Fig 1:**
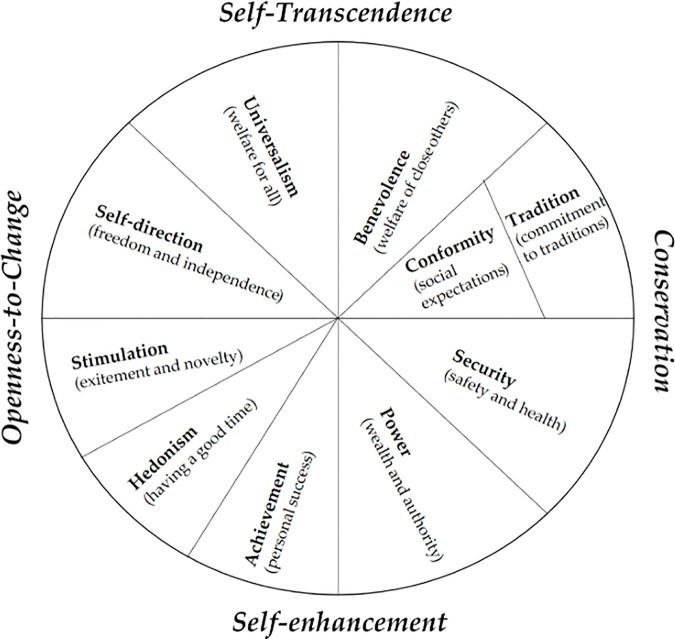
The value circle as described in the theory of basic human values. Source: [26].

Schwartz and Bilsky [15] used the RVS as a starting point to construct a novel theory underlying human values and their relation to each other. According to this theory, human values developed from three basic needs, i.e., biological needs, interaction requirements, and societal demands which can be categorized as having personal or social focused goals and either anxiety-avoidance or growth motivation. Using Smallest-Space Analysis (a form of non-metric MDS), they showed that human values form a circular structure of relations among values, such that values closer around the circle are more positively correlated than distant values [23]. This structure had important implications, in that external variables (e.g., attitudes, behavior) were correlated with the values according to the circle. Specifically, any external variable tends to have similar correlations to neighboring values in the circle, while correlations with values opposite in the circle are in the opposite direction. This insight greatly enhances our understanding of values as an evaluative system which is related to phenomena by a value’s relative importance within the system and organized through opposing motivational forces (growth-anxiety) and non-compatible goal contents (personal-social) (Fig 1).

From this seminal work came the Schwartz Values Survey (SVS [23]), validated in 20 countries. The first versions of the SVS included all the items of the RVS, as well as new items to increase coverage of the circle (e.g., power, universalism – nature, tradition), and rating as the measurement scale. Additionally, all items were accompanied with a short explanation in parentheses, e.g., “FREEDOM (freedom of action and thought)”.

Further improvements, additions and alternative versions of the measurement instrument [17,27] showed the value circle could also be observed using other methods. For example, the Portrait Values Questionnaire (PVQ) [18] is less abstract and uses a rating scale. The PVQ includes 40 short descriptions of a person (instead of single words) and asks respondents to compare themselves with the person in the description. This exercise greatly reduces cognitive load, compared to evaluating abstract concepts such as “freedom”, and is currently the most used instrument.

Over time, various versions of the PVQ developed: using fine-grained division of the human values [28], a picture based [29] and video clip based [30] versions for children, as well as a short version with 21 items for the European Social Survey [31,32], a Best-Worst measure [19], and an 11-item version for the World Values Survey [33] among others. The circular structure emerges using any of these questionnaires. Thus, while the measurement method has undergone numerous refinements over the years, the assessment of the human values construct appears to be feasible using diverse metrics.

### The RVS in the LISS panel

There are distinct differences between the original RVS and the RVS instrument used in the LISS. It should be noted that the RVS was developed and used primarily in the U.S.A. and English-speaking countries and was not translated to other languages. This may create challenges when applying the RVS in a different cultural or linguistic context, as is the case with the LISS, which is conducted in Dutch [34]. Consequently, some translations of the RVS items in the LISS can be ambiguous or lose their original meaning. For instance, the original RVS item “Family Security” is simply translated to “de familie” (i.e., “the family”) in Dutch, dropping any reference to security. The item “inner harmony” is translated literally but it is not a common phrase or concept in Dutch language or culture, thus losing its intended significance. The results of the current study are therefore mainly applicable to the RVS version used in the LISS.

Furthermore, despite the clear legacy of the RVS in the SVS, interpreting and comparing the results of the two instruments can be challenging. While the RVS presented words for participants to rank, such as “Freedom”, the SVS also added a clarification in parenthesis, “Freedom (freedom of action and thought)”. The term “Freedom” shown to participants in the RVS without the qualifier “(freedom of action and thought)” as shown in the SVS, carries a broad range of potential meanings and implications depending on the respondent’s perspective. Additionally, some RVS items appear in the SVS as the main item and other times in parenthesis behind the main item. Thus, although both instruments use similar items, there is no clear one-to-one correspondence.

Given the limitations of the RVS, we first conducted a theory-based assessment, matching the items to the descriptions of the value types as proposed by Schwartz [23]. After excluding unsuitable items, we assigned each RVS item to a provisional Schwartz value type, presented in Table 2. We then assess whether the RVS instrument in the LISS aligns with the quasi-circumplex value structure defined by Schwartz using multidimensional scaling. Finally, we validated the resulting value indexes by regressing them on socio-demographic variables, key value-related personality traits, and political orientation. These results will allow researchers to establish a consistent and updated framework for assessing human values in a longitudinal panel useful to many researchers, thereby improving the potential for meaningful comparisons and insights across different studies.

**Table 2:** Rokeach value survey items, their translations and fit to Schwarz instrument and theoretical value type.

Dutch item	Authors’ translationa	Similar items in SVS or PVQ-21	Theoretical Schwartz (1992) value type
eerlijk, oprecht	Fair/honest, sincere	SVS – Honest	Benevolence
verantwoordelijk	responsible	SVS (excluded, BE/CO/UN)	Benevolence or Conformity^b^
hard werkend	hard working	SVS - Ambitious (hardworking, aspiring)	Conformity, Achievement, or Self-direction
vergevingsgezind	forgiving	SVS - forgiving	Benevolence
open	Open could be applied to people or ideas.	SVS – Broadminded	Universalism/ Benevolence
moedig	Courageous, meaning depends highly on context.	None	Achievement/Self-direction/ Stimulation
behulpzaam	helpful	PVQ-21 – helpful	Benevolence
liefdevol	Loving	none	Benevolence/ Universalism
competent	Competent but multiple interpretations	SVS – Capable	Achievement
netjes	Tidy, neat, proper.	PVQ-21 – behave properly	Tradition or Conformity
gedisciplineerd	Disciplined, possible interpretation as self-directed.	SVS – Self-discipline	Self-direction (disciplined in following own goals) or Conformity
onafhankelijk	independent	PVQ-21 – independent	Self-direction
vrolijk	Cheerful, upbeat, happy	none	Excluded, emotion
beleefd	Polite	SVS – politeness	Conformity
intellectueel	Intellectual	SVS – intelligent excluded (SD/UN/CO)	Achievement
gehoorzaam	Obedient	PVQ-21 – follows rules	Conformity
logisch, consistent	Logical, consistent.	SVS – intelligent (logical, thinking) excluded (SD/UN/CO)	Achievement/ Self-direction
creatief	Creative	PVQ-21 – Creative	Self-direction
wereldvrede	World peace	SVS – a world at peace	Universalism
het gezin	The family	SVS – excluded (CO/BE/AC)	Excluded. Ambiguous meaning
vrijheid	Personal freedom	SVS – freedom	Self-direction
gelijkheid	Equality	PVQ-21 – equality	Universalism
zelfrespect	Self-respect	SVS Excluded (AC/UN/BE/SE)	Excluded – not a value: attitude
geluk	Luck or happiness or good fortune	none	Excluded – not a value: concept or emotion
wijsheid	Wisdom	SVS – Wisdom	Universalism
nationale veiligheid	National security	PVQ-21 – strong government	Security
verlossing	Salvation	SVS – Devout	Excluded. Religious or spirituality item^c^
ware vriendschap	True friendship	Excluded from SVS (BE/UN/SE/TR/SD/AC)	Benevolence
prestatie	Achievement	successful in PVQ-21	Achievement
innerlijke harmonie	Inner harmony	Excluded from SVS (BE/SE/TR/SD/ST)	Universalism
comfortabel leven	A comfortable life	none	Hedonism
liefde en seksualiteit	Love and sexuality	Excluded from SVS (BE/UN/SE/CO/TR)	Excluded. Unclear meaning.
schoonheid	Beauty	SVS – a world of beauty	Universalism
plezier	Pleasure, having fun	Pleasure in PVQ-21	Hedonism/ Stimulation
erkenning, status	Recognition, status	Excluded from SVS (PO/AC/SE)	Power/ Achievement.
opwindend leven	An exciting life	Exciting life in PVQ-21	Stimulation

*Note.* UN = universalism, BE = benevolence, CO = conformity, TR = tradition, SE = security, AC = achievement, PO = power, HE = hedonism, ST = stimulation, SD = self-direction. Documentation on the question wording and items is available here: https://www.dataarchive.lissdata.nl/concepts/view/87.

^a^Three native Dutch speaking value experts translated the items for the present research, discrepancies in translation were discussed.

^b^Responsibilities can be part of a position or social role that creates a relationship between two strangers and conformity values would be applicable here in carrying these out. ^c^ Religious and Spirituality items are a separate dimension [23,54].

## Materials and methods

### Participants

The data for this study come from two sources. The main source is the longitudinal internet study for social sciences (LISS), an online panel of Dutch households managed by the non-profit research institute Centerdata [4]. The panel was established in 2007 with a representative random sample of 10,000 households drawn from population registers by Statistics Netherlands. Since then, the recruitment of replenishment samples maintained the representativeness and size of the panel. An external ethics committee assesses each questionnaire. Participants are asked for informed consent during recruitment (see https://www.lissdata.nl/ethics). Panel members completed online questionnaires on a variety of topics each taking about 15–30 minutes in total every month and received a small fee for each completed questionnaire. The RVS is asked annually. Additionally, the LISS includes cross-sectional questionnaires by sampling the longitudinal panel participants. One of these cross-sectional questionnaires was the World Value Survey (WVS) [35] which included an 11-item version of the PVQ (PVQ-11).

The second source of data is the Dutch sample of the European Social Survey [36]. The ESS is a probability-based and nationally representative cross-sectional survey. The ESS was established in 2001 and conducted bi-annually. The ESS subscribes to the Declaration on Professional Ethics of the International Statistical Institute, which includes informed consent, while the ESS ERIC Research Ethics Board assesses questionnaires.

We used these data sources to conduct an exploratory analysis and assess whether the indexes coincide with established value measurement instruments. For exploratory analysis, we used the first wave of the LISS personality module, the 2008 wave [34] (N = 6768) as it was the first in the LISS panel and therefore contains a representative sample of the Dutch population.

In the assessment, we compared the ESS, the WVS and the LISS longitudinal panel data. Each dataset has strengths and weaknesses. The ESS is a high-quality data source with a validated measurement, a 21-item version of the PVQ, (PVQ-21) developed by Schwartz [37]. In contrast, the WVS administered the PVQ-11 which is known to have some weaknesses, and in particular, the coverage of the value circle is limited [38]. Thus, differences between the PVQ-11 and the RVS results could be due to covering different facets of the same value, while this should be less of an issue with the PVQ-21 from the ESS. A weakness of the ESS is that it is an independent sample and survey, thus discrepancies with the LISS data could be due to methodological differences. The WVS, on the other hand, was administered through the LISS and therefore contains some of the same participants who went through an identical procedure to answer the PVQ-11 and the RVS.

The WVS was collected in 2012 and to minimize confounding factors, we chose waves from the LISS and ESS that were collected around the same time. Additionally, to facilitate comparison across samples and data sources, we restricted the sample age range from 25 to 67. We chose this age range as there are greater changes in values earlier in life while in middle adulthood value change slows down [5,39,40]. These less stable periods may affect relationships with socio-demographic variables. The World Value Survey (WVS) of 2012 [41] includes 1,901 observations and 1,383 after excluding cases of missing values and restricting the age range. In the LISS wave 2013 (N = 5,169) and after excluding cases of missing values and restricting the age range, there were 3,651 cases. Lastly, the ESS wave 2012 (N = 1,845) includes 1,321 cases after case selection.

### Measures

In the LISS version of the Rokeach Value Survey, participants are asked to rate each of the values as guiding principles in their lives on a scale from 1 “Very unimportant” to 7 “Very important”. Each value was presented as a single word; see Table 2 for the list of items.

The Portrait Value Questionnaire (PVQ-21) is a 21-item measure of human values [31] deployed in the European Social Survey. The PVQ-21 consists of short verbal portraits of different people. For each item, respondents indicate how similar the person described is to themselves on a 6-point scale ranging from “very much like me” to “not like me at all”. An example of an item is: “It is important to him to be rich. He wants to have a lot of money and expensive things”. A shorter version, the PVQ-11, used in the WVS, is identical except that it includes only one item per value type and two items for the universalism value.

Data from the RVS, PVQ-11 and PVQ-21 were ipsatized, that is, the mean rating of all items was subtracted from each item, as recommended by Schwartz [17]. Ipsatization adjusts scores for differences in scale use and more accurately reflects the theoretical construct of values as guiding behavior through their relative importance within the value system of an individual. There are strong theoretical and empirical reasons for using ipsatization [42–44].

The mean rating of the RVS items within respondents used for its assessment was computed using the formula (1) to ensure that the mean rating was not biased by the greater number of items pertinent to some values, like universalism and benevolence (4–5 items), relative to others, such as conservation and self-direction (2–3 items). We computed the average of i to n items belonging to each of j to k values, then computed the average of the values. The mean rating was computed for each respondent p in each year y of data collection.


$${Mean\;rating}_{py}=\;\frac{\sum_{k}^{j}\frac{\sum_{n}^{i}x_{i}}{n}}{k}$$

The LISS and ESS data also include education which was standardized to the Statistics Netherlands categories of primary school, lower secondary (VMBO), higher secondary (HAVO/VWO), college (HBO) and university (WO, reference category). The gender variable was coded as 0 for women and 1 for men.

### Data Analysis

The data analysis was conducted in three phases. First, we selected suitable items in the RVS given their theoretical (face-value) fit and empirical location on MDS projections. Second, we confirmed the assigned value-types of the selected items using MDS. Third, we compared the rating and relationship with other variables of values indexes in the LISS in comparison to the ESS and WVS data.

Therefore, we first conducted a theory-based assessment, matching the value items to the descriptions of the value types. After excluding unsuitable items, we assigned each RVS item to a provisional value type. Then we entered the items into a weak confirmatory multidimensional scaling (MDS) analysis [45] to explore which items empirically fit to their provisionally assigned value type.

Multidimensional scaling is a method for visualizing the similarity or dissimilarity of a set of objects in a low-dimensional space. Exploratory forms of MDS often use random starting configurations. Weak confirmatory MDS imposes a starting configuration on the variables. In both cases, the MDS finds the optimal configuration of points by minimizing a stress function, which is an indicator of how well the data are represented in the resulting figure (a smaller stress indicator suggests a better representation of the data in the figure). Using weak confirmatory MDS, researchers test the fit of a model to the data, while allowing some flexibility to adjust to the observed similarities.

In our case, we used the ordering of value types in the theoretical representation of the value circle [23] to assign each item coordinates on the unit circle. Additionally, to obtain robust results, we split our data into ten sub-samples using non-replacement random sampling of the LISS wave 2008. An item was accepted as representing a value type if it appears in the majority of MDS plots in the area of its assigned position and did not induce high stress. The stress-I statistic is a measure of model fit, calculated by the difference in the distance between positions between pairs of items in the low-dimensional space and their estimated similarities (correlations).

All MDS analyses were conducted in R4.3.2 using the Smacof 2.1–5 package [46,47] with a maximum iteration of 1x10^40^ and a convergence criterion of 1x10^-40^. Following Schwartz et al. [17]we used pairwise observations to estimate correlations as measures of similarity and transformed these to dissimilarities by subtracting them from one. The MDS analyses were ordinal, meaning a non-metric function was used to compute disparities, which preserve the rank order between objects.

After the final item selection, we calculated the scales using the LISS 2013 wave. We conducted four analyses to confirm the similarity of these indexes. First, we conducted a weak confirmatory MDS to examine whether the quasi-circumplex structure of values emerges. Second, we computed the rank of values by using the sample means of the ipsatized ratings. We assessed the ranking of values using items from the RVS by comparing them the PVQ-11 (WVS) and PVQ-21 (ESS). Third, we assessed the coordinates of a weak confirmatory MDS of the RVS to those of the PVQ-21, using Procrustes analysis. Fourth, to assess the similarity between instruments by using demographic variables to predict value indexes from the RVS, PVQ-11 and PVQ-21, showing all indexes have similar relationships

We used circular MDS on the final selection of RVS items in the 2013 LISS wave, as well as those in the PVQ-11 (WVS) and the PVQ-21 (ESS). This algorithm forces items towards positions on the unit circle depending on the penalty parameter, which we set to 100 to ensure items would fall on the unit circle but also induces stress, lowering model fit. In all figures in text and the supplementary materials we show the stress-I statistics of the weak confirmatory and the circular MDS.

To compare the MDS solutions across samples we used Procrustes analysis. Procrustes analysis works by rotating, scaling, and translating one configuration to match another, while minimizing the sum of squared differences between the corresponding points. Tucker’s congruence coefficient quantifies their similarity [45], where coefficients higher than 0.95 indicate equal configurations [48].

To assess the similarity of our instruments we compared the relationships of the values with several socio-demographic predictors. We chose socio-demographics, as societal structures and distinct life experiences can contribute to varying value preferences [49–51]. We compared the value indexes by estimating standardized regression coefficients using the RVS, PVQ-11 and PVQ-21 indexes as a dependent variable and estimating the effects of age (continuous), gender and education level (nominal). Additionally we examine the value’s relationship with Left-Right political orientation, and two personality traits, extraversion and intellect for which sufficient evidence exists of their relationship [42] or specifically in the Dutch population [52], as the correlation between values and attitudes is contingent on both dispositional and situational factors [53].

## Results

### Choosing suitable items from the RVS

We first examined all items for theoretical (face) validity. Three native Dutch speaking value experts translated the items for the present research, discrepancies in translation were discussed. Each item was assigned to a value using definitions found in Table 1 and information in Schwartz et al. [23]. The theoretical allocations of RVS items to the values are presented in Table 2, column 4. A few items were unsuitable for measuring basic human values. These items were either too ambiguous in Dutch, or they did not fit the definition or contents of values put forward by Schwartz [23]. We excluded the following items: love and sexuality, family, salvation, happy, happiness and self-respect. Justifications for their exclusion can be found in Table 2, column 4.

Table 2 also includes a note on whether a similar item occurs in other value instruments in the third column. Many RVS items were integrated into the SVS or portrait descriptions of the PVQ-21. Other RVS items were excluded in the construction of the SVS [23] due to their unstable location in the MDS projections. If an item was excluded in Schwartz [23], this is also noted in the fourth column of Table 2, and the locations of these items in the MDS conducted by Schwartz are listed.

### Assessing empirical location of face-valid items in the RVS

Fig 2 shows the coordinates of the weak confirmatory MDS using the first subsample of the LISS 2008 wave. The Procrustes rotation with the other subsamples showed that the congruence coefficients were 0.96 or above, indicating the solutions were very similar. In Table 3, we list all items plotted with the number of times they appear in each region of the MDS space. We noted any patterns in their location and recurrent stress contributions. A recurring pattern of a high proportion of stress contributed which indicates its location does not fit with the theoretical structure imposed by the weak confirmatory MDS. All the MDS figures using ipsatized scores (Figures 1–10 in S1 Figs), the total stress per item (Table 1 in S2 Tables) and heat maps of the stress per item (Figures 11–20 in S1 Figs) are in the supplementary materials. The same MDS analyses using the ratings of values can be found in the supplementary material (Figures 21–30 in S1 Figs).

**Fig 2:**
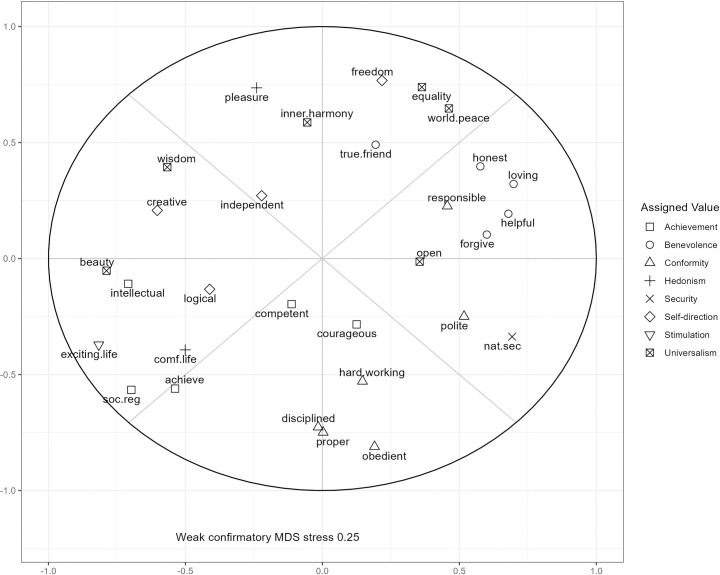
Coordinate plot of selected ipsatized Rokeach Value Survey items in the LISS in sub-sample 1 using assignments listed in Table 2.

**Table 3:** Number of times an item is observed in expected location (in brackets) in weak confirmatory MDS in ten subsamples of the LISS 2008 wave and notes on inconsistencies.

Label	Value location
Honest	Benevolence (10)
Responsible	Benevolence (10)
Hard working	Conformity (10)
Forgiving	Benevolence (10)
Open	Universalism (1)/ Benevolence (9)
Courageous	Achievement (1)/Self-direction (1)/ Stimulation (1)/ **Conformity** (8)
Helpful	Benevolence (9)/ Conformity (1)
Loving	Benevolence (9)/ Universalism (1)
Competent	Achievement (2)/ **Conformity** (3)/ Unclear (5)
Proper	Tradition (0)/ Conformity (10)
Disciplined	Self-direction (0) or conformity (10)
Independent	Self-direction (2)/Achievement (2)/ Unclear (6)
Polite	Conformity (10)
Intellectual	Achievement (10)
Obedient	Conformity (10)
Logical	Conformity (0)/ Self-direction (0)/ **Achievement(5)/** unclear (5)
Creative	self-direction (3)/ **Stimulation (4)/** Achievement (3)
World peace	Universalism (9)/ **Benevolence(1)**
Freedom	Self-direction (0)/ **Universalism (10)**
Equality	Universalism (10)
Wisdom	Universalism (0)/ Self-direction (10)
National security	Security (0)/ Universalism (4)/ Unclear (6)
True friendship	Benevolence (0)/ **Universalism (10)**
Achievement	Achievement (10)
Inner harmony	Universalism (8)/ Stimulation(2)
A comfortable life	Hedonism (5)/ Universalism, or Stimulation (5)
Beauty	Stimulation (10)
Pleasure	Unclear (10)
Recognition, status	Power (0)/ Achievement (10)
An exciting life	Stimulation or Hedonism (10)

*Note.* Higher numbers in parenthesis indicate a more stable item. Value locations in bold are unexpected.

Overall, Fig 2 shows clear and distinct clusters of self-direction, benevolence, and universalism and conformity items while items on achievement, stimulation and hedonism were mixed. As a result, the following items were excluded due to their incongruent positions, high contributions to stress and/or ambiguous interpretations: hard working, courageous, competent, independent and logical-consistent. Additionally, the following items were re-categorized due to their location on the MDS plot: wisdom (self-direction), beauty (stimulation), true friendship (universalism) and freedom (universalism).

Lastly, considering the trade-off between completeness and model fit, there are some items that we kept despite a high stress or appearance in unexpected quadrants: pleasure, national security, a comfortable life, and creative. We retained these items as they have a clear theoretical location and maintain a good coverage of the value circle. Our observations and considerations on these items are discussed below.

### Considerations on self-direction item “creative”

The “creative” item was unstable in its MDS location across ten sub-samples and contributed a considerable amount of stress to the model. However, we found the stress and likely the MDS location to be induced by a strong correlation with an item that did not make the final selection, namely “logical and consistent” (labelled as logical in Fig 2). Therefore, the stress induced by this item in the exploratory MDS is of less importance. The “creative” item is also well-established and included in the SVS and PVQ-21 as a self-direction item. Therefore, we opted to include it in the final selection.

We note that the division between “wisdom” as a universalism item or a self-direction item is unclear from the MDS projections. We have opted to categorize it as a self-direction item due to its strong negative correlation with all the conformity items. We believe it reflects autonomy of thought – which according to Schwartz [17] should be negatively correlated with conformity items and close to universalism, as it is close to intellectual openness and considering other’s points of view.

### Considerations on security item “national security”

National security is a salient item for respondents of many countries, but less so in the Netherlands. It is likely that this item is interpreted in many fashions and may reflect a political inclination rather than a value. This item contributed by far the most stress and had inconsistent locations across the MDS projections of the ten sub-samples. Nevertheless, it is an item that is clearly pertinent to the security-societal value and maybe a useful item in certain research contexts. Therefore, we include it for completeness, but caution against its use as a good indicator of the security value within the Dutch population.

### Considerations on stimulation and hedonism items “pleasure”, “comfortable life”, “exciting life” and “beauty”

As shown in Fig 2 and noted in Table 3, the items for hedonism and stimulation were difficult to interpret as they were often found close together in the exploratory MDS. This pattern is not unusual, as the items of these values are often intermixed in the SVS [23]. Based on the detailed definitions of these values in Schwartz et al. [17], we decided to categorize the items “pleasure” and “comfortable life” as hedonism and the items “exciting life” and “beauty” as stimulation items. Hedonism is described as the pursuit of sensuous gratification with low arousal, which comes with an avoidance of the stress and competition associated with achievement and power values. The “comfortable life” fits this description and is in an appropriate location for a hedonism item in Fig 2, in between the stimulation and achievement items. Stimulation is described as an orientation towards arousal and interest, thus the item “exciting life” fits as a stimulation item. As expected from the definition of stimulation and its location on the value circle, “exciting life” and “beauty” have positive correlations with achievement items and negative correlations with benevolence items.

Additionally, the “pleasure” item has the expected negative correlations with conformity items given hedonisms position on the opposite side of the value circle. However, the separation of the two hedonism items from stimulation content is less clear. First, the “comfortable life” item is positively correlated with achievement and the “exciting life” item. Second, the “pleasure” item is correlated with both the other hedonism item “comfortable life” and the stimulation item “exciting life”. Moreover, the “pleasure” item is often found among the self-direction items in the MDS projections. Therefore, although we choose to separate these items into stimulation and hedonism, an argument could be made to merge these four items into a broader stimulation-hedonism measurement, or to use cross-loadings to capture both hedonism and stimulation components.

### Final assignment of value items to values

**Fig 3:**
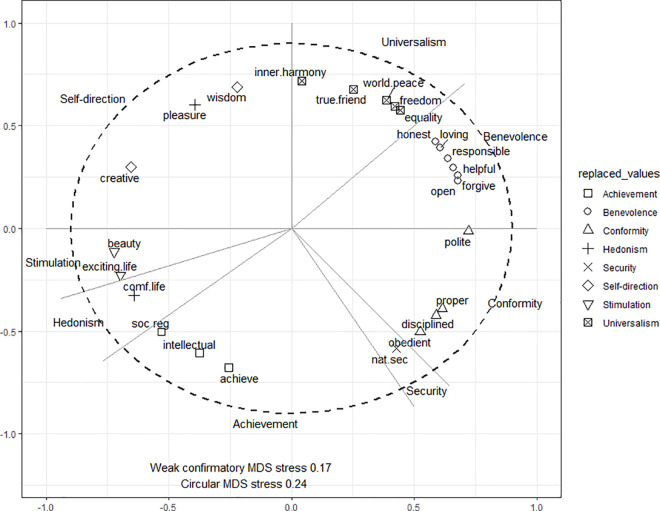
Circular Weak Confirmatory MDS of the Selected RVS items. *Note.* Based on LISS wave 2013. Items are ipsatized by mean rating.

Table 4 shows the final item assignments based on the weak confirmatory MDS results with their variable number in the LISS data. Fig 3 shows a weak-confirmatory circular MDS analysis using the ipsatized items in Table 4 using data from LISS wave 2013. The expected quasi-circumplex structure of value types emerges clearly using the subset of items, the Stress-I value of the circular solution, 0.23, is significantly lower (p < .001) than random permutations, confirming a good fit. All value types are in the expected order around the circle. Only one item, “pleasure” (measuring the hedonism value) is not located in its expected area, and is among self-direction items. Additionally, the security item “national security” is found in the theoretically expected position between conformity and achievement items. However, the “national security” item still contributes a disproportionate amount of stress (17% of the total), which reduces the model fit.

**Table 4:** Recommended Items and Value Assignment.

Value	Best fit	Cronbach’s Alpha [CI 95%]	Possible Items (with lower reliability)
Universalism	A world at peace (117), Equality (120), Freedom (119)	0.57 [0.56 - 0.59]	Inner harmony (128), True friendship (126)
Benevolence	Honest (99), Forgiving (102), Helpful (105), Loving (106), Open (103)	0.62 [0.61 - 0.64]	Responsible (100)
Stimulation	Beauty (131), An exciting life (134)	0.42 [0.39 - 0.45]	
Self-direction	Creative (116), Wisdom (123)	0.18 [0.15 - 0.22]	
Hedonism	A comfortable life (129)		Pleasure (132)
Achievement	Intellectual (113), Achievement (127), Social recognition (133),	0.48 [0.45 - 0.50]	
Power	None		
Security			National security (124)
Conformity	Obedient (114), Polite (112), Proper (108)	0.46 [0.43 - 0.48]	Disciplined (109)
Tradition	None		

*Note*. The number between parentheses indicates the item-number in the LISS archive. UN = universalism, BE = benevolence, CO = conformity, TR = tradition, SE = security, AC = achievement, PO = power, HE = hedonism, ST = stimulation, SD = self-direction.

Based on the results shown in Fig 2 and Fig 3, we categorized items into two groups. The first group includes best fitting items based on their stability in the MDS solutions, face-validity and positive contribution to Cronbach’s alpha. Using the best fitting items, the Cronbach’s alpha of all indexes are moderate to low, comparable to those previously reported for the SVS [Schwartz, 2005] and PVQ-21 [Schwartz, 2003]. However, the self-direction index has a poor Cronbach alpha, likely because the items span two distinct facets (thought and behavior) [17], which may impact its correlation with other variables of interest. The second group of items have high face validity but lack a stable MDS location (pleasure), induce a high stress (national security), or have a negative impact on Cronbach’s alpha (responsible, disciplined, true friendship and inner harmony). Lastly, we report the fit statistics of a confirmatory factor analysis (CFA) in the online supplementary material Table 20 of S2 Tables for all values with at least 3 items assigned as ‘best fitting’ and one CFA combining the stimulation and hedonism items. Note that we did not find suitable items for power and tradition values (Fig 3).

### Assessing relationships between RVS, PVQ-21, PVQ-11, attitudes and demographics

#### Procrustes rotation with the PVQ-21 and PVQ-11.

The circular MDS solutions of indexes constructed using the RVS items were compared with MDS solutions of the indexes of two original Schwartz value instruments using Procrustes rotation. This procedure showed that configuration of the eight value types found in the RVS 2013 data were highly similar to the configuration of the same values in the ESS 2012 wave measured by the PVQ-21. The congruence coefficient was 0.97 indicating that the two configurations are very similar (Fig 32 in S1 Figs).

#### Ranking of items using RVS, PVQ-21 and PVQ-11.

The ranking of values is quite stable within populations [Schwartz and Bardi, 2001]. If the three questionnaires measure comparable values, their ranking in the same population should also be similar. As can be seen in Table 5, ranking of values in the RVS, PVQ-21 and PVQ-11 are similar. The only large difference between the data sources is the position of the self-direction value, which is ranked higher in the PVQ-21 than the PVQ-11 and RVS. We inspected each item to understand the differences in ranking. The high ranking of self-direction in the PVQ-21 is due to the “ Important to make own decisions and be free” item which receives the highest mean ipsatized score. The other self-direction item in the PVQ-21, “creative”, is ranked third similar to cross-national findings [55]. The PVQ-11 and RVS, which both include the creative item, rank it in fifth place. Thus, when comparing the same items, the self-direction value is still higher ranked in the PVQ-21 than in the other two instruments. Corroborating these findings we calculated the ranking of values in separate age groups which provided similar results (Table 3–10 in S2 Tables). Overall, the ranking of values using the RVS seems very similar to ranking of values computed by using the PVQ-21 and PVQ-11 instruments, but it is important for future users to bear in mind that the aspects of freedom and independence are absent from the self-direction index in the LISS.

**Table 5:** Rankings of Values in the Netherlands in Ipsatized indexes.

Value	LISS 2013 RVS	WVS 2012 PVQ-11	ESS 2012 PVQ-21
Benevolence	1	1	1
Self-direction	5	5	2
Universalism	2	2	3
Security	3	3	4
Hedonism	4	6	5
Conformity	6	4	6
Tradition	NA	7	7
Achievement	7	8	8
Stimulation	8	9	9
Power	NA	10	10

*Note*. LISS is the Longitudinal Internet Study for Social Sciences. WVS = World Value Survey, ESS = European Social Survey. RVS = Rokeach Value survey. PVQ = Portrait Value Questionnaire.

#### Relationship of RVS values with attitudes and personality traits.

In this section we test the utility of the RVS value indexes by leveraging the circumplex structure of values to examine their relationship with left-right political orientation and two personality traits, namely agreeableness and intellect. The strength of relationship between a value and another variable should change with the positioning of the value on the circle due to the compatibilities and incompatibilities of value’s goal content and motivation [23].

Van Herk et al. [52] found that politically left-leaning respondents tend to prioritize universalism, benevolence and self-direction while right-leaning respondents prioritize security, conformity, and achievement. Using the RVS in the LISS data, we find similar relationships where benevolence (r = − 0.14, p < .001), universalism (r = − 0.22, p < .001) and self-direction (- 0.16, p < .001) are associated with left-wing responses while conformity (r = 0.17, p < .001) and achievement (r = 0.06, p < .001) are associated with right-wing responses on the left-right orientation item. These correlations indicate that the RVS indexes are associated with left-right political orientation as found in van Herk et al. [52] and follow the pattern predicted by the theory of basic human values.

Parks-Leduc et al. [42] conducted a meta-analysis to examine relationships between values and personality traits. They found the agreeableness trait is strongly positively related to universalism, benevolence, conformity and tradition while intellect is associated with self-direction, stimulation and universalism values. The RVS indexes in the LISS data show similar patterns of relationships. Agreeableness has a substantive correlation with universalism (r = 0.22, p < .001), benevolence (r = 0.22, p < .001) but was unrelated to the conformity index (r = − 0.00, p = .96). As expected, due to the circumplex structure of values, agreeableness was also negatively associated with stimulation (r = − 0.13, p < .001) and achievement (r = − 0.20, p < .001). Intellect is associated with self-direction (r = 0.26, p < .001) weakly correlated with universalism (r = 0.04, p < .001) and unrelated to stimulation (r = − 0.02, p = .113). As expected from the circumplex structure, intellect is also related to benevolence (r = 0.08, p < .001) and tradition (r = − 0.14, p < .001). Associations between personality traits and values were not all replicated; however, the theoretically most similar values did have a correlation while the theoretically opposing values were also negatively related. Thus, providing evidence that the value measures show expected relationship with similar constructs and their relationships follow theoretical structure of values. Table 19 in S2 Tables shows a correlation matrix of the RVS value indexes with all big-five personality traits, the left-right orientation, attitudes regarding marriage, divorce, immigration and multicultural society.

#### Relationship with demographic variables.

Demographic characteristics such as age, gender and socio-economic status can reliably predict the relative importance of values. To assess similarities between value indexes using RVS items and the PVQ-21 and PVQ-11, we show the standardized regression coefficients of age, gender and education level predicting each of the eight value types. Since all three instruments measure similar value constructs, we expect them to exhibit similar relationships in their respective representative samples of the Dutch population.

Previous studies using cross-sectional samples have shown that the social focused values (benevolence, universalism, conservation, tradition, security) are positively associated with age while person focused values (achievement, power, self-direction and stimulation) are negatively associated with age [49,56–60]. Past research has also shown that gender is associated with the importance of benevolence and achievement [51,61]. In general, women value benevolence more than men, and achievement values less than men. Lastly, higher socio-economic position is associated with lower importance of conservation and security, and higher importance of stimulation and self-direction values [50,53,62–64]. In the regressions we use education level as a proxy for socio-economic position.

Reporting our results, we follow the theoretical structure of the Schwartz value circle, and report the results of 8 of the 10 values for which we had sufficiently fitting items in the RVS (i.e., excluding tradition and power). All reported coefficients are standardized and significant at the p < .01 level unless otherwise noted. All regression tables can be found in the supplementary materials Tables 11–18 in S2 Tables.

When comparing our three measurement instruments, benevolence has similar relations to gender in all three data sources as expected, although varying in strength using the RVS (β = − 0.25, S.E. = 0.03) and the PVQ-11 (β = − 0.50, S.E. = 0.05). As expected from previous research, age is positively related to benevolence in the PVQ-11 (β = 0.17, S.E. = 0.03), and RVS but not significantly related in the PVQ-21 (β = 0.05, S.E. = 0.03, p = .094).

As expected, the RVS conformity index was related to education level, with secondary educated respondents rating conformity higher (β = 0.53, S.E. = 0.06) compared to the reference category of university educated respondents. These relationships fit the findings of sociological research [64,65] which has extensively shown that higher educated individuals and those with complex jobs value conformity less than the lower educated. However, the RVS index of conformity did not have a statistically significant relationship to age (β = − 0.02, S.E. = 0.02, p = .22), which was unexpected given a documented positive relationship in most other samples, including the PVQ-21 (β = 0.17, S.E. = 0.03). Closer inspection of the RVS conformity items showed that the item “proper” had a correlation with age. This item also features in the PVQ-21 index of conformity. Note that the PVQ-11 conformity index also had a non-significant coefficient with age (β = − 0.04, S.E. = 0.03, p = .16) – despite also asking about proper behavior. Therefore, the relationships with demographic variables of the conformity items seems to fit previous findings but indicate that the RVS items may capture different aspects of conformity values.

The security value in the RVS is based on the “national security” item. It is associated with gender a similar manner in the RVS (β = − 0.21, S.E. = 0.05), PVQ-11 (β = 0.11, S.E. = 0.06) and PVQ-21 (β = − 0.22, S.E. = 0.03, p = .04) and with secondary educated compared to university educated respondents in the RVS (β = 0.30, S.E. = 0.08), PVQ-11 (β = 0.47, S.E. = 0.12) and PVQ-21 (β = 0.44, S.E. = 0.12) as expected from literature cited previously. We found a positive relationship with age in the RVS (β = 0.12, S.E. = 0.02) and PVQ-21 (β = 0.11, S.E. = 0.03) but not in the PVQ-11 (β = 0.03, S.E. = 0.03, p = .38).

The achievement value seems to have a similar relationship with demographics across the three instruments. It was negatively related to age in the RVS (β = − 0.06, S.E. = 0.03), PVQ-21 (β = − 0.20, S.E. = 0.02) and PVQ-11 (β = − 0.20, S.E. = 0.03). However, the strength of the effect was much lower with the RVS index. All three indexes showed that men prioritise achievement more than women do, as expected from previously cited literature in the RVS (β = 0.31, S.E. = 0.03), PVQ-21 (β = 0.20, S.E. = 0.03) and PVQ-11 (β = 0.29, S.E. = 0.05).

The hedonism indexes were negatively associated with age in the RVS (β = − 0.25, S.E. = 0.02), PVQ-21 (β = − 0.21, S.E. = 0.03) and PVQ-11 (β = − 0.17, S.E. = 0.03), as expected. All three indexes showed a positive effect of secondary compared to university education in the RVS (β = 0.16, S.E. = 0.08), PVQ-21 (β = 0.25, S.E. = 0.12) and PVQ-11 (β = 0.35, S.E. = 0.13).

The stimulation (ST) indexes had a similar and expected relationship with age in the RVS (β = − 0.15, S.E. = 0.02), PVQ-21 (β = − 0.11, S.E. = 0.03) and PVQ-11 (β = − 0.11 S.E. = 0.03), and with gender in the RVS (β = 0.36, S.E. = 0.03), PVQ-21 (β = 0.17, S.E. = 0.05) and PVQ-11 (β = 0.41, S.E. = 0.06) and secondary education compared to university education in the RVS (β = − 0.15, S.E. = 0.08, p = .05), PVQ-21 (β = − 0.37, S.E. = 0.03) and PVQ-11 (β = − 0.16, S.E. = 0.12, p = .19) although this effect was not significant in the PVQ-11.

The self-direction indexes had different associations with age and gender between samples but were similarly related to education, as expected, in all three samples. The PVQ-21 index had no significant relationship with age (β = 0.03, S.E. = 0.03, p = .32) or gender (β = 0.07, S.E. = 0.05, p = .22), while RVS index was significantly predicted by age (β = 0.09, S.E. = 0.02) and gender (β = 0.10, S.E. = 0.03). Lastly, the PVQ-11 index was predicted by gender (β = 0.22, S.E. = 0.03) but not by age (β = − 0.01, S.E. = 0.03, p = .79). As expected, the secondary educated respondents prioritize self-direction less than university educated in the RVS (β = − 0.27, S.E. = 0.08), PVQ-21 (β = − 0.30, S.E. = 0.12) and PVQ-11 (β = − 0.28, S.E. = 0.13).

The universalism (UN) index in the RVS had similar relationships with socio-demographics as the PVQ-21 and PVQ-11 indexes in the theoretically expected direction. In all three datasets, universalism had a positive relation with age in the RVS (β = 0.21, S.E. = 0.02), PVQ-21 (β = 0.19, S.E. = 0.03) and PVQ-11 (β = 0.32, S.E. = 0.03) and a negative relation with the male gender in the RVS (β = − 0.39, S.E. = 0.03), PVQ-21 (β = − 0.30, S.E. = 0.05) and PVQ-11 (β = − 0.28, S.E. = 0.05). Overall, the expected relationship with age and gender indicate the universalism index in the RVS is consistent with other measurement instruments.

## Discussion

This paper established indexes based on items from the Rokeach Value Survey (RVS) to measure values from the Schwartz’s [23] theory of basic human values using the publicly available LISS panel data, thereby opening a new avenue for researchers to examine the relationship between human values and a broad range of attitudes, traits, and behaviors. We show that the RVS items in the LISS can be meaningfully interpreted following the theory of basic human values as conceived by Schwartz [23]. We show a theoretically informed selection of RVS items form the value circle in a circular MDS, indicating the correlation structure between items follows the theorized structure between values. The ranking of the indexes in the respondents aged 25−65 is almost identical in the PVQ-21, PVQ-11 and RVS, except for the self-direction value, which is unusually highly ranked in the ESS PVQ-21. Finally, the relations with demographics of the value indexes in the RVS of the LISS data are very similar to the established PVQ-21 in the ESS data, and the relationships to the most value-laden personality traits and to political orientation as found in previous research.

There are some limitations of the RVS indexes in the LISS data: while some values are well represented and have at least three items available with a clear theoretical value type, a stable MDS location and similar relationships with other variables (universalism, achievement, benevolence, conformity), other values are less well represented with only one or two items (self-direction, stimulation, hedonism). Nevertheless, these value indexes have clear theoretical value types and showed similar relationships with demographics, personality traits, and political orientation as other measurements. The only exception is the security measure which has one item (national security) with an inconsistent location on the exploratory MDS plots of all 32 RVS items. However, this item did present itself in the expected location in the confirmatory MDS with the subset of RVS items and showed similar relationship with demographic variables. Unfortunately, a limitation of the RVS is that it lacks suitable items to measure power and traditional values. Additionally, the rating scale used in the LISS makes it incomparable to ranking versions of the RVS. Thus, the indexes developed here should only be used for RVS versions that use a comparable rating scale like the LISS.

The present findings suggest that measurements of values based on the theory of basic human values can be produced using instruments derived from different theories. To quantify to what extent the two instruments probe one common mental representation of a psychological concept, we suggest future research uses data containing information from the same participants recorded in the target instruments to establish scalar equivalence between the RVS and a reliable Schwartz measurement instrument.

This paper re-interprets secondary data in view of theoretical advances. This approach is not unique in the literature regarding efforts to link outdated instruments from secondary data with the modern Schwartz values framework. Smallenbroek et al. [39], for example, found that the value instrument from the German Socio-Economic Panel (GSOEP) from Kluckhohn values theory could be used to reproduce the Schwartz value circle adequately for a longitudinal investigation into the lifespan development of human values. Borg et al. [66] showed that the instrument developed in the values theory of Helmut Klages, could be used to reproduce the Schwartz values framework. Efforts linking past and present research methodologies allow reinterpretation of secondary data and can greatly enhance our understanding of human values by facilitating comparisons between studies thereby facilitating knowledge accumulation.

We derive general recommendations for future research using the LISS data. Researchers interested in testing hypotheses on specific human values may use the LISS data to reproduce universalism, achievement, benevolence and conformity. Self-direction, stimulation and hedonism may likewise be reproduced, but with the limitation that these are captured with one or two-items only. Furthermore, researchers interested in testing hypotheses about higher-order human values should note that in some cases, for example, self-enhancement, the value’s motivational goals are only partially covered.

We hope this paper will facilitate more longitudinal research on personal values and their relationships to the many other traits, attitudes and behaviors measured in the LISS data. To help other researchers implement the indexes presented we have provided SPSS, R and Stata syntax in the “S3 Code” supplemntary materials file. We hope that these will maximize cumulative knowledge by creating a standardized measurement of values in the LISS data for future studies, aligning it with the measurement instruments of the theory of basic human values.

## Supporting information

S1 Fig MDS Projections, Heatmaps, and Procrustes Rotation Figures.
(ZIP)

S2 Tables Stress Per Item, Procrustes Rotation Congruence Coefficient, Value Rankings, Regression Tables, Correlation Matrix with Attitudinal Questions, Model Fit Table of the Confirmatory Factor Analysis.
(DOCX)

S3 Code SPSS, R and Stata Syntax for Computing Value Scales.
(DOCX)
