# Supplementary figures and images for: Constructing Schwartz values framework using the Rokeach values survey: Human value measurement in the longitudinal internet survey for social sciences
Source: PLoS One. 2025 Aug 12;20(8):e0329179. doi: 10.1371/journal.pone.0329179 (PMC12342246; doi:10.1371/journal.pone.0329179)

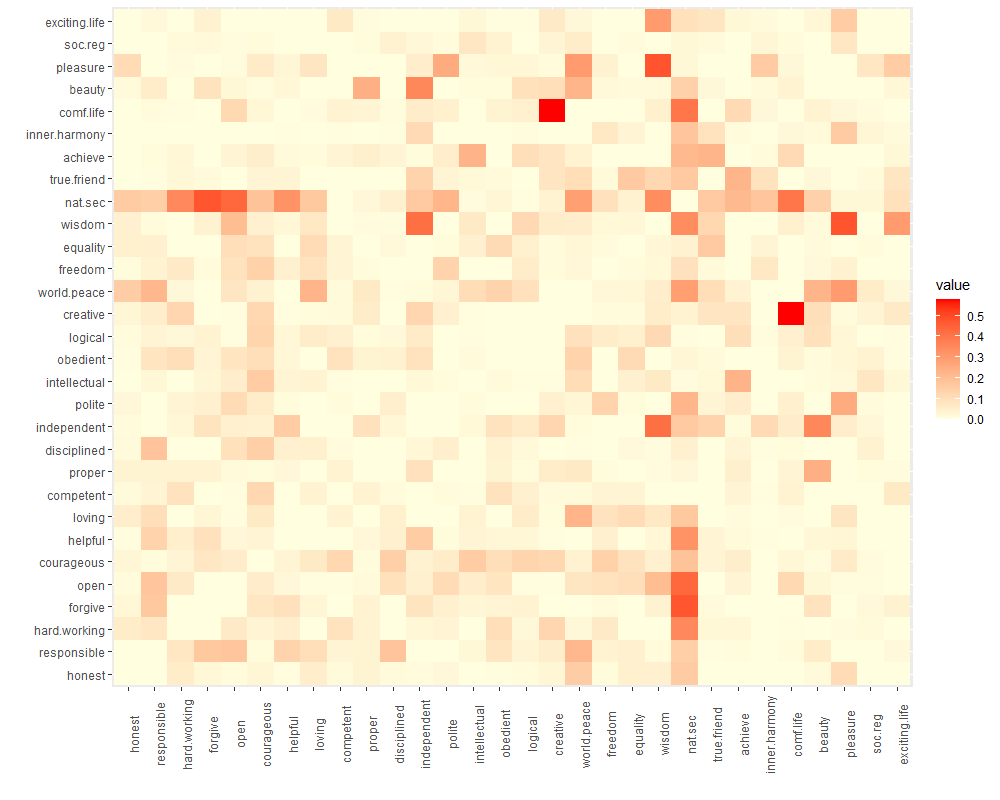

Supplement: S1 Fig — MDS Projections, Heatmaps, and Procrustes Rotation Figures. (ZIP) [file pone.0329179.s001.zip › Renamed Files/Fig12.tif]

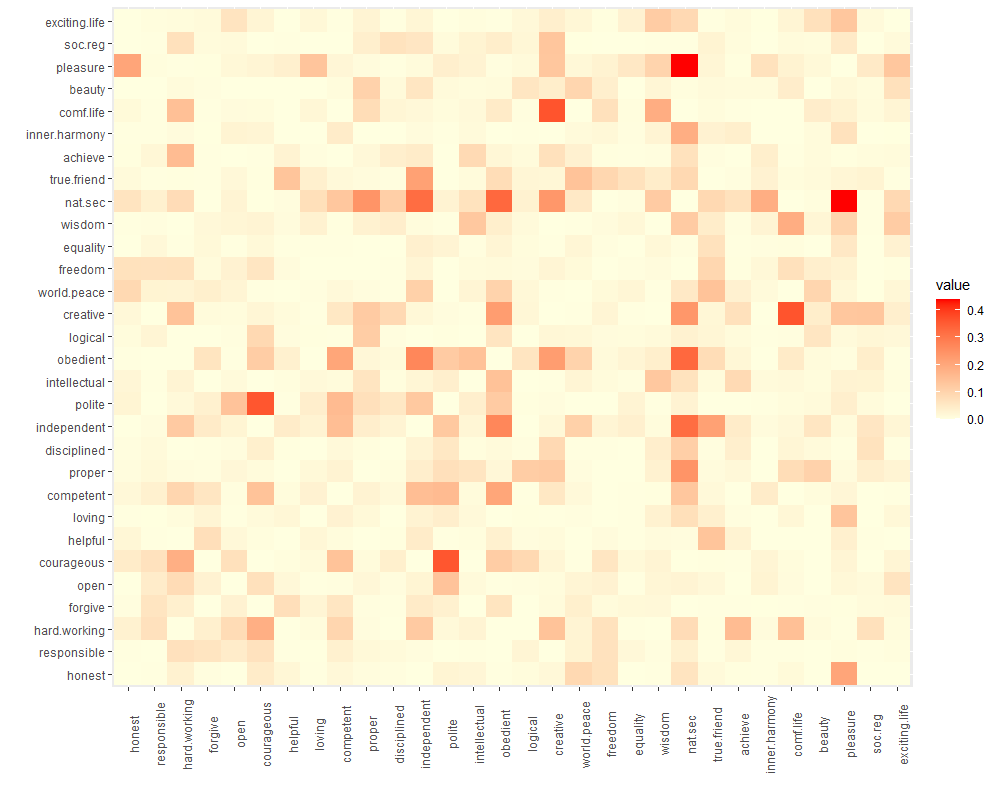

Supplement: S1 Fig — MDS Projections, Heatmaps, and Procrustes Rotation Figures. (ZIP) [file pone.0329179.s001.zip › Renamed Files/Fig13.tif]

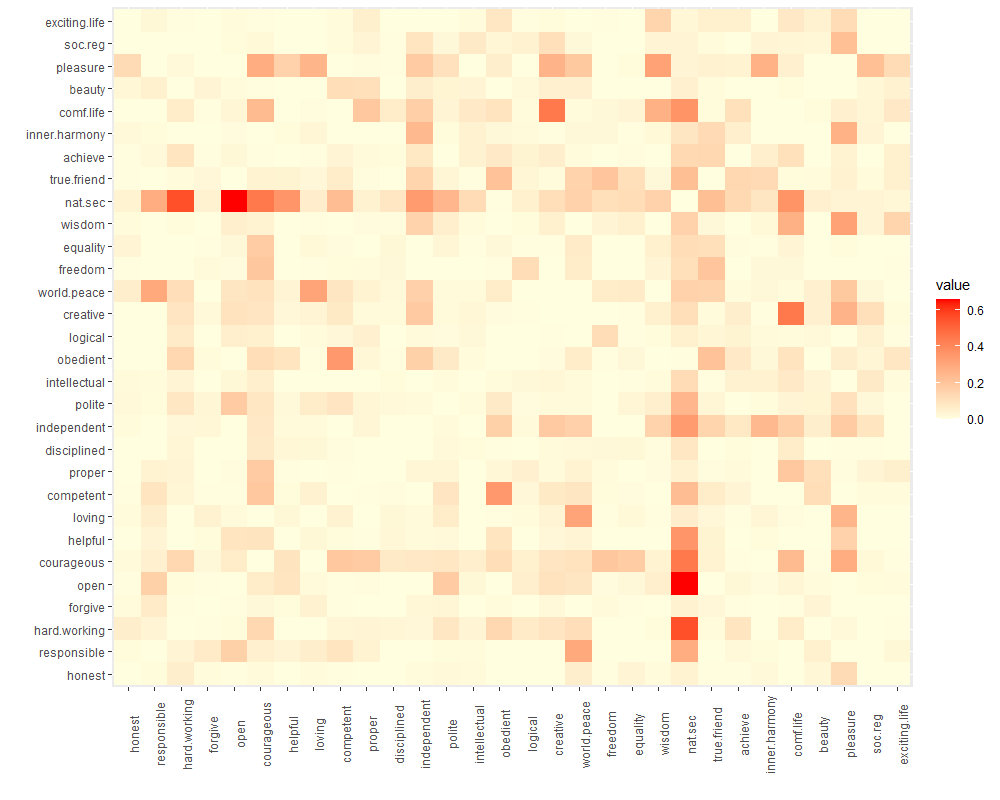

Supplement: S1 Fig — MDS Projections, Heatmaps, and Procrustes Rotation Figures. (ZIP) [file pone.0329179.s001.zip › Renamed Files/Fig14.tif]

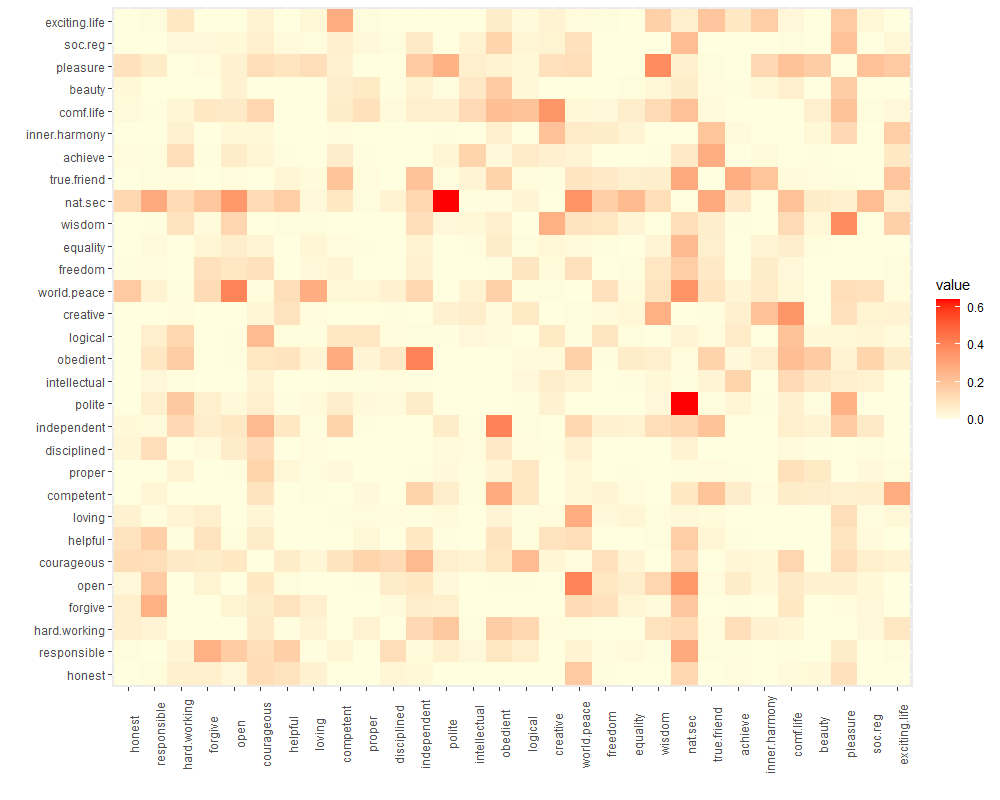

Supplement: S1 Fig — MDS Projections, Heatmaps, and Procrustes Rotation Figures. (ZIP) [file pone.0329179.s001.zip › Renamed Files/Fig15.tif]

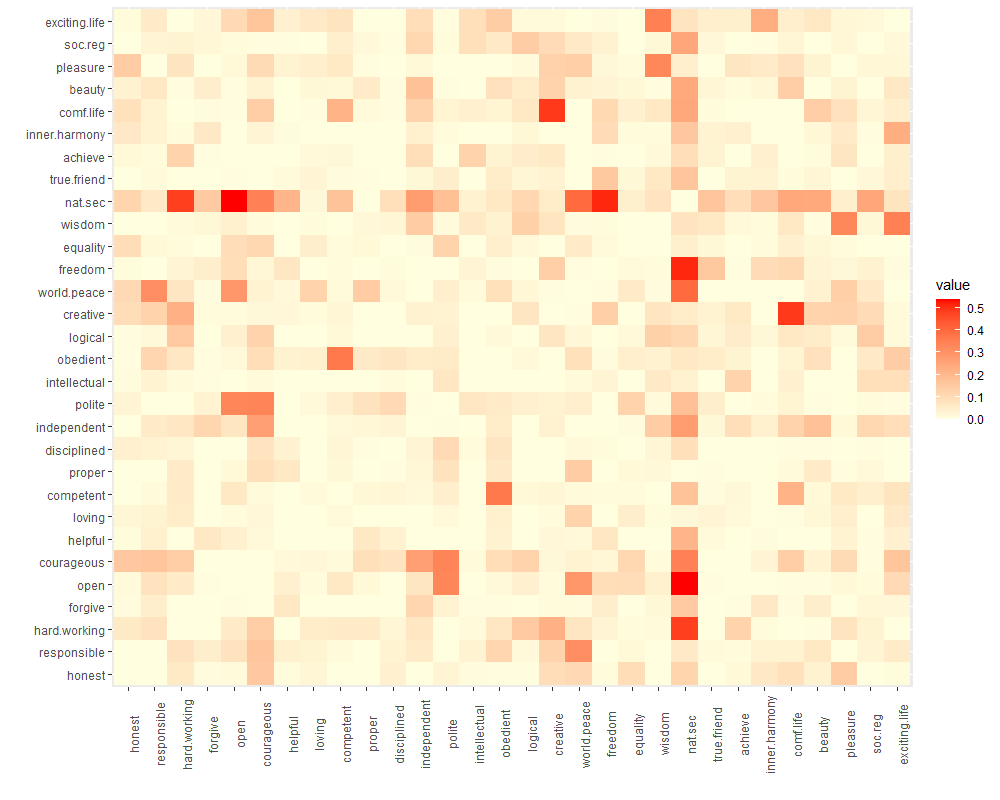

Supplement: S1 Fig — MDS Projections, Heatmaps, and Procrustes Rotation Figures. (ZIP) [file pone.0329179.s001.zip › Renamed Files/Fig16.tif]

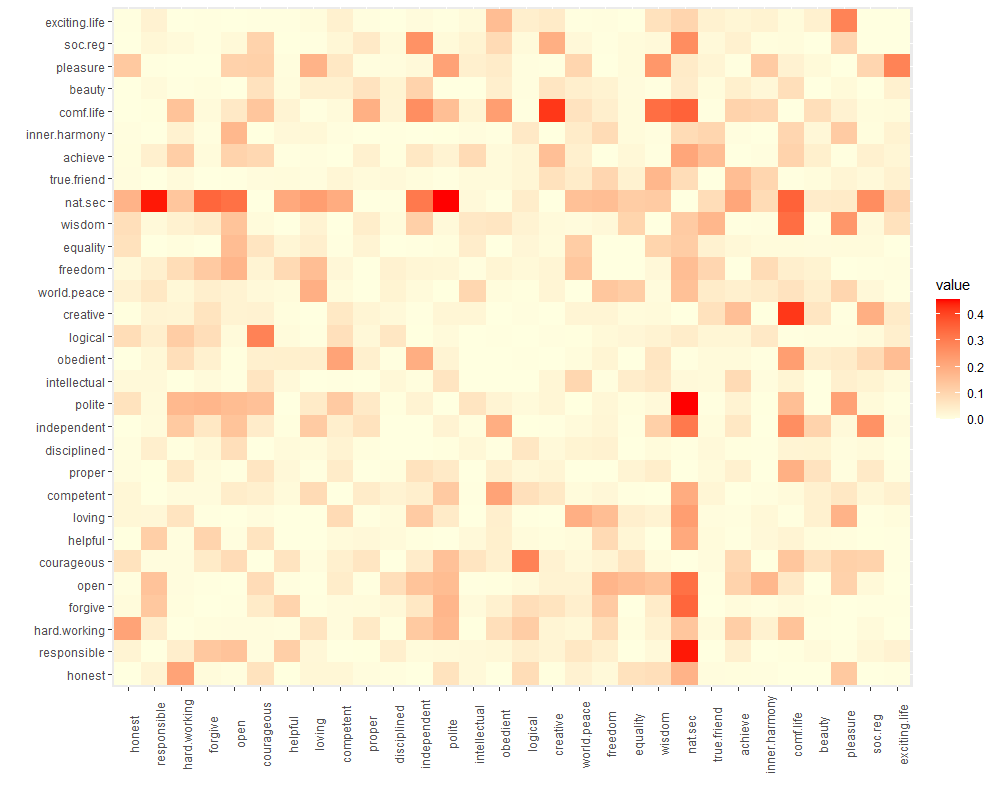

Supplement: S1 Fig — MDS Projections, Heatmaps, and Procrustes Rotation Figures. (ZIP) [file pone.0329179.s001.zip › Renamed Files/Fig17.tif]

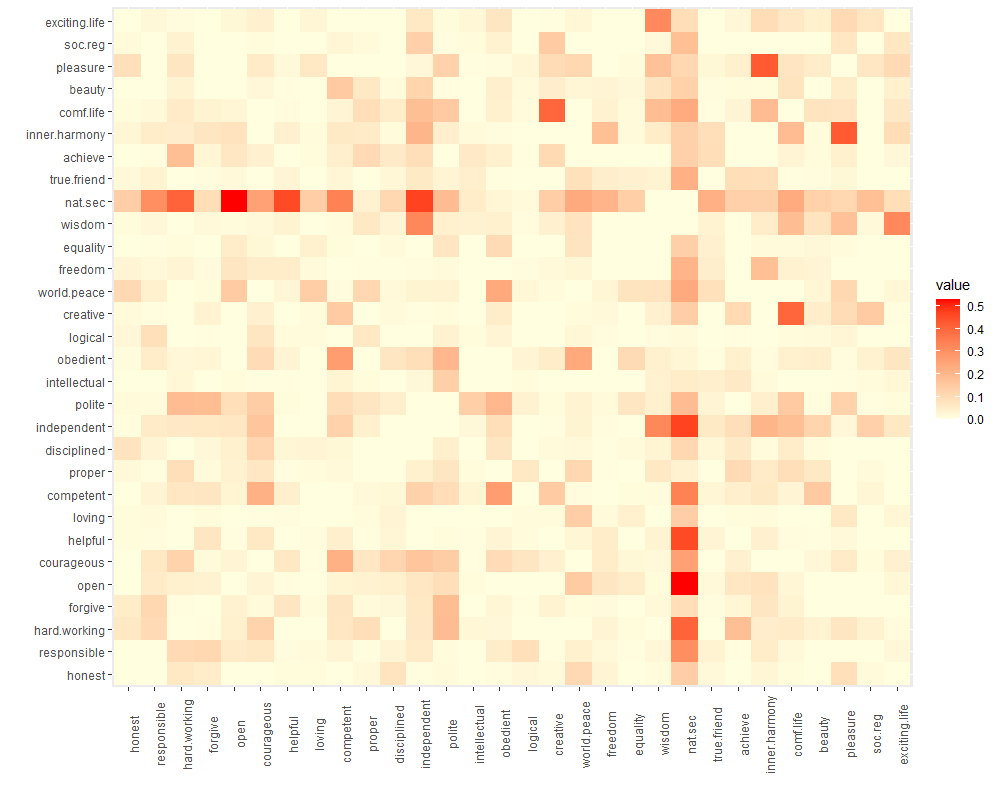

Supplement: S1 Fig — MDS Projections, Heatmaps, and Procrustes Rotation Figures. (ZIP) [file pone.0329179.s001.zip › Renamed Files/Fig18.tif]

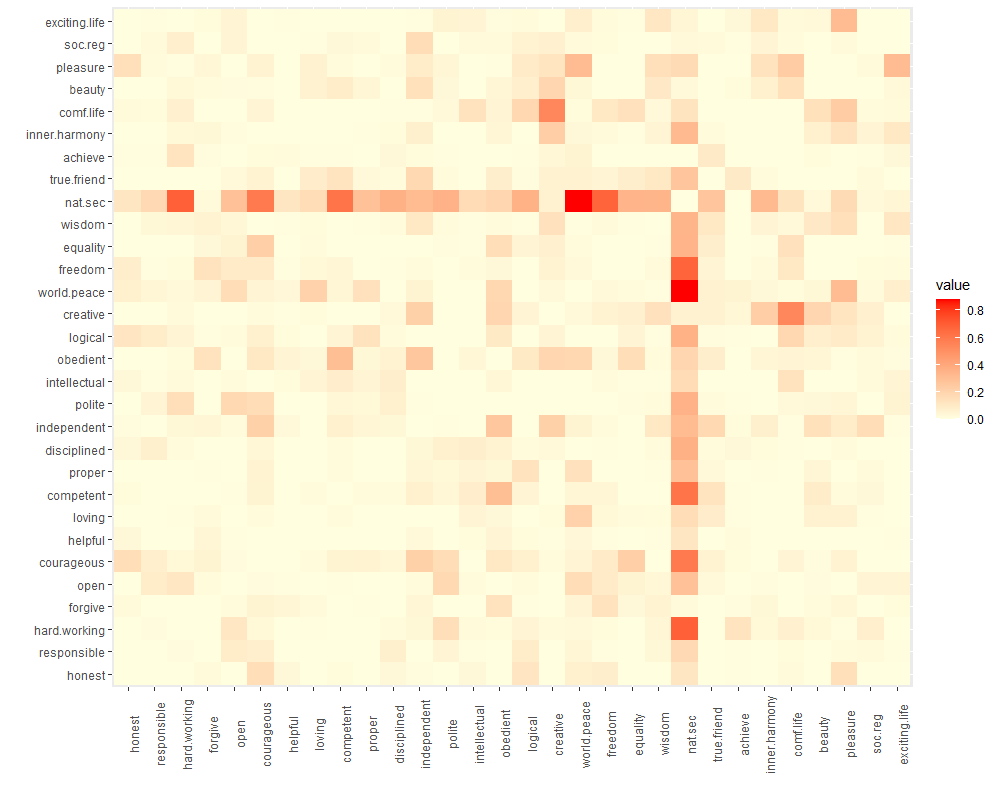

Supplement: S1 Fig — MDS Projections, Heatmaps, and Procrustes Rotation Figures. (ZIP) [file pone.0329179.s001.zip › Renamed Files/Fig19.tif]

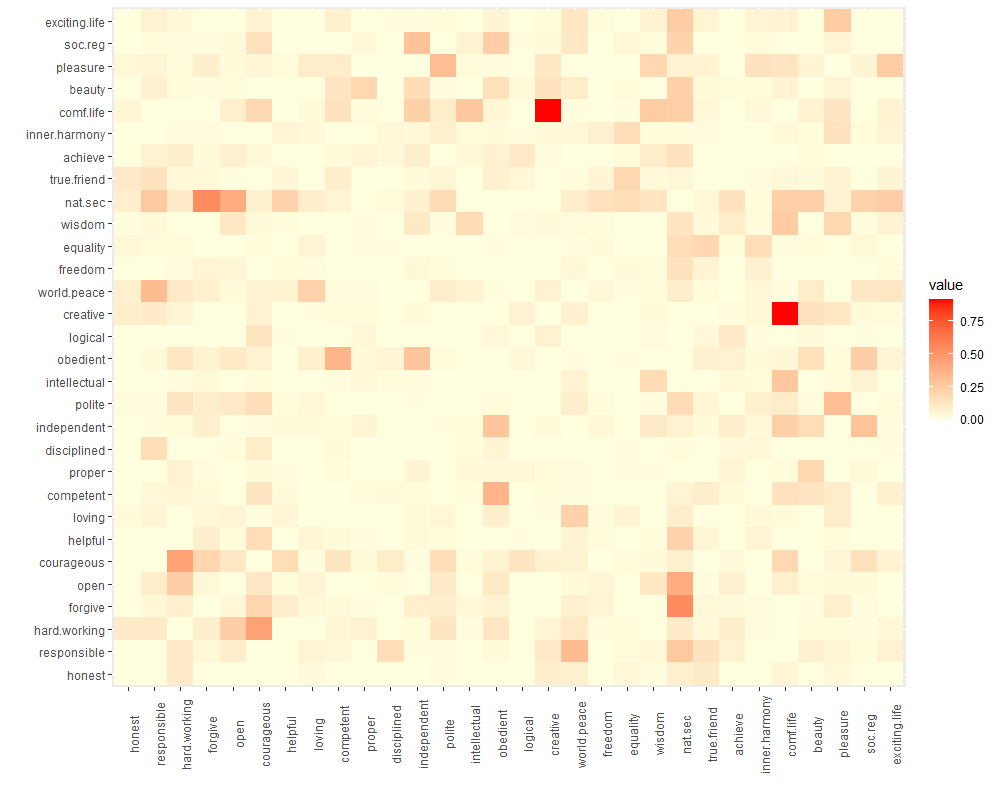

Supplement: S1 Fig — MDS Projections, Heatmaps, and Procrustes Rotation Figures. (ZIP) [file pone.0329179.s001.zip › Renamed Files/Fig20.tif]

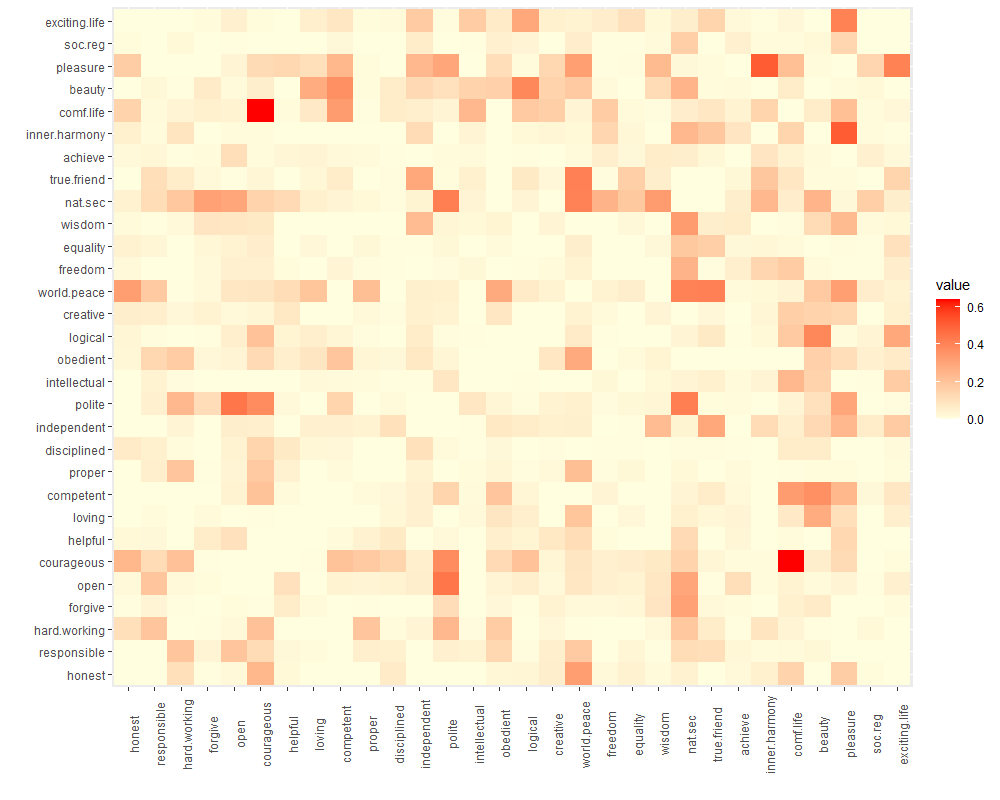

Supplement: S1 Fig — MDS Projections, Heatmaps, and Procrustes Rotation Figures. (ZIP) [file pone.0329179.s001.zip › Renamed Files/Fig11.tif]

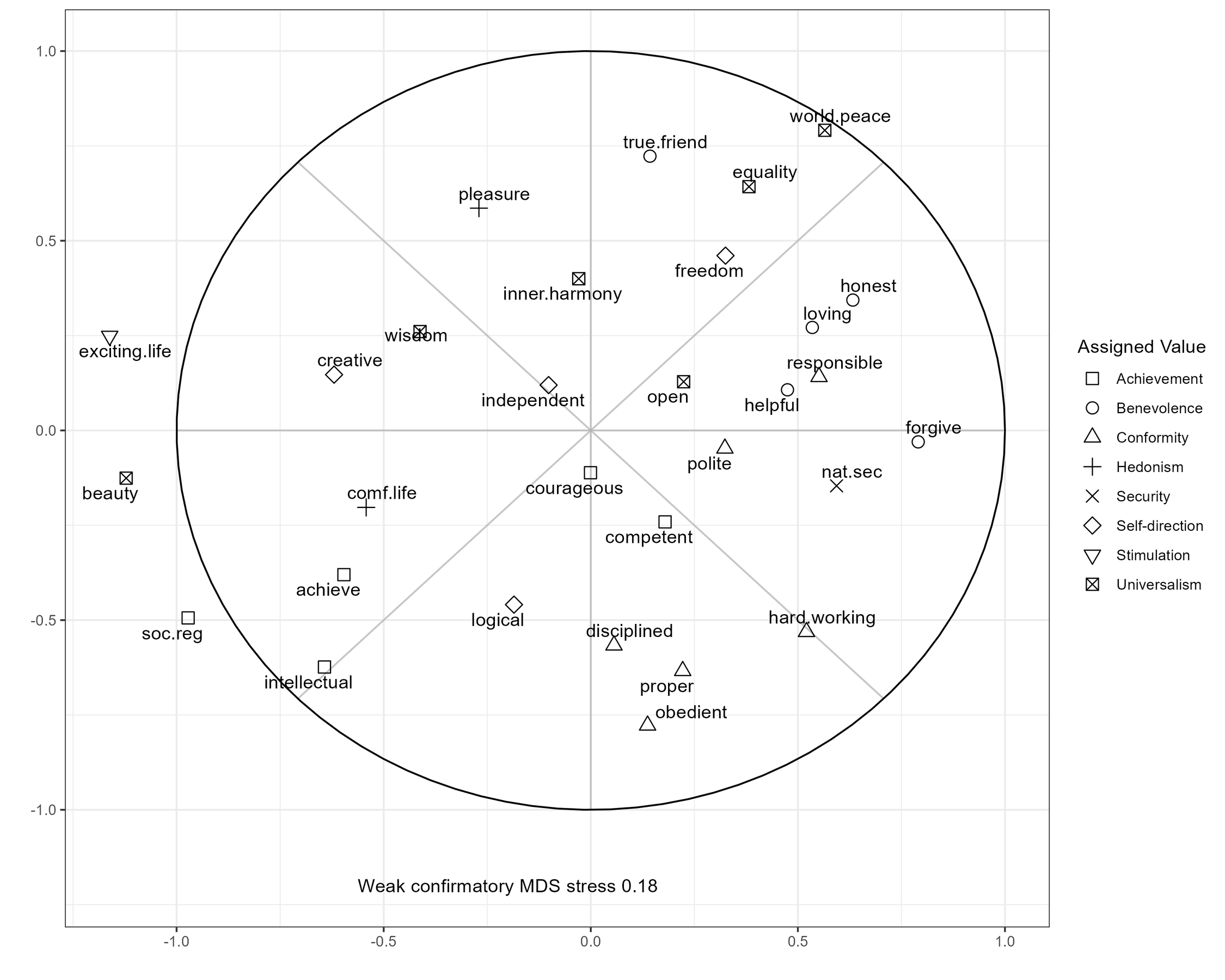

Supplement: S1 Fig — MDS Projections, Heatmaps, and Procrustes Rotation Figures. (ZIP) [file pone.0329179.s001.zip › Renamed Files/Fig26.tif]

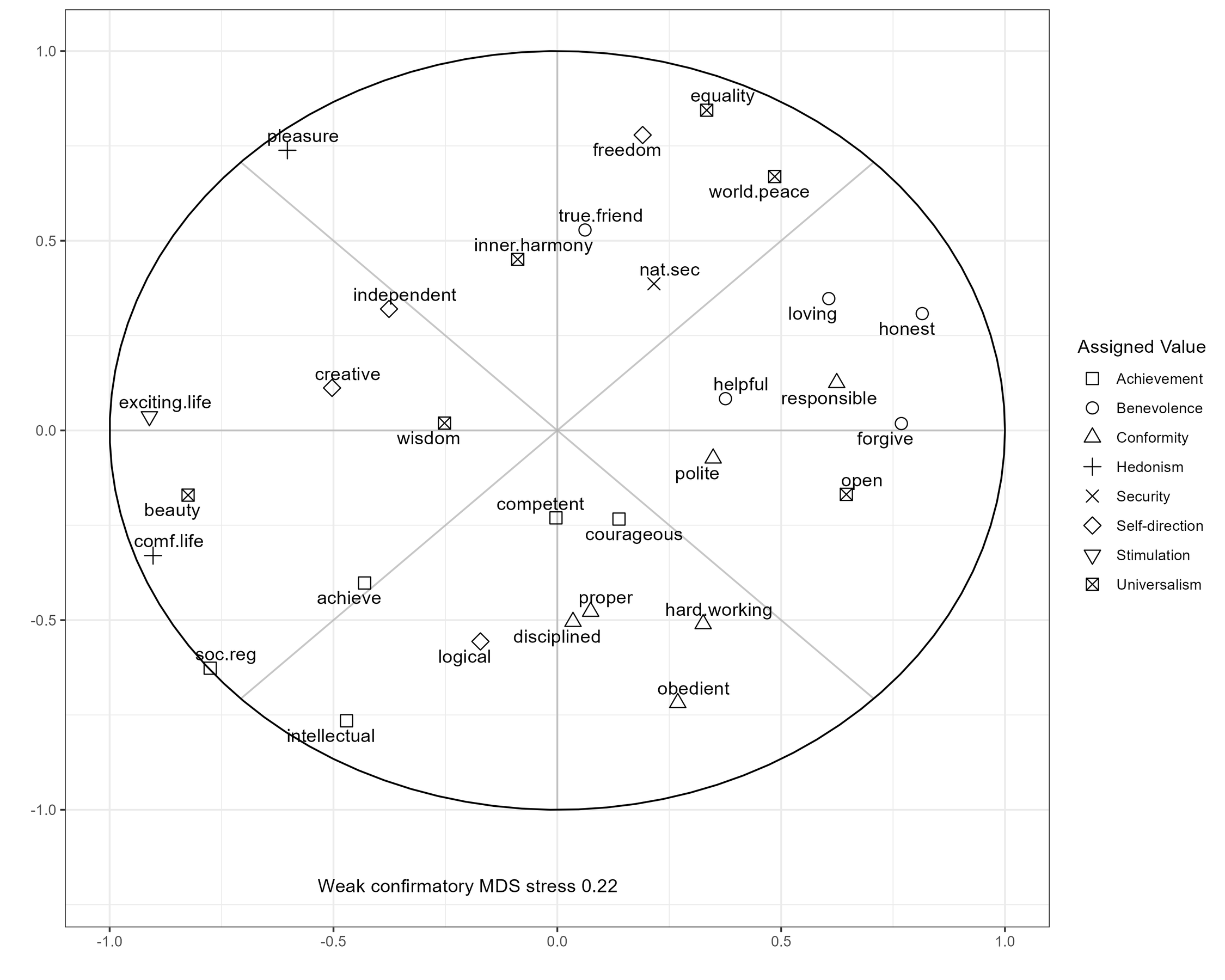

Supplement: S1 Fig — MDS Projections, Heatmaps, and Procrustes Rotation Figures. (ZIP) [file pone.0329179.s001.zip › Renamed Files/Fig23.tif]

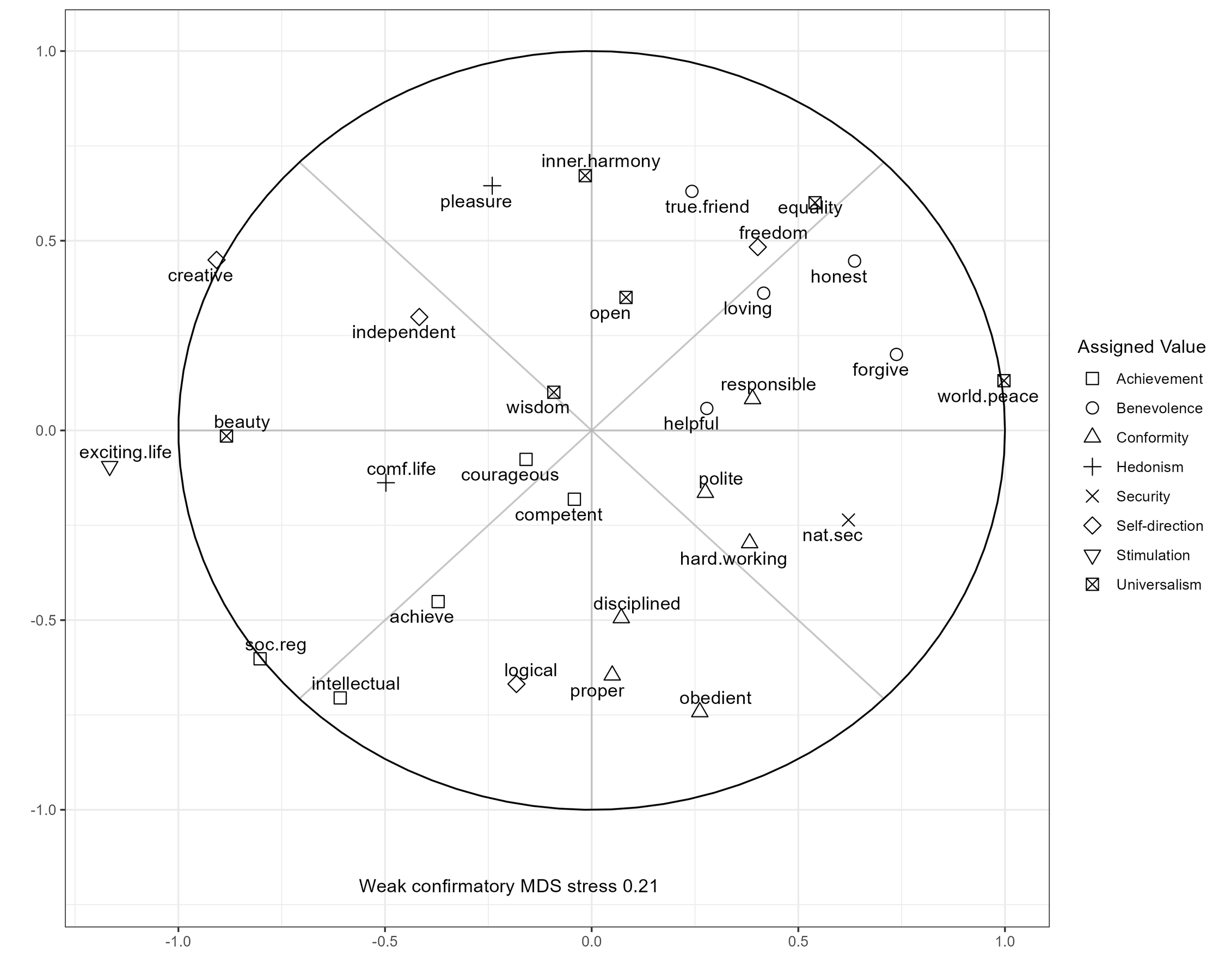

Supplement: S1 Fig — MDS Projections, Heatmaps, and Procrustes Rotation Figures. (ZIP) [file pone.0329179.s001.zip › Renamed Files/Fig24.tif]

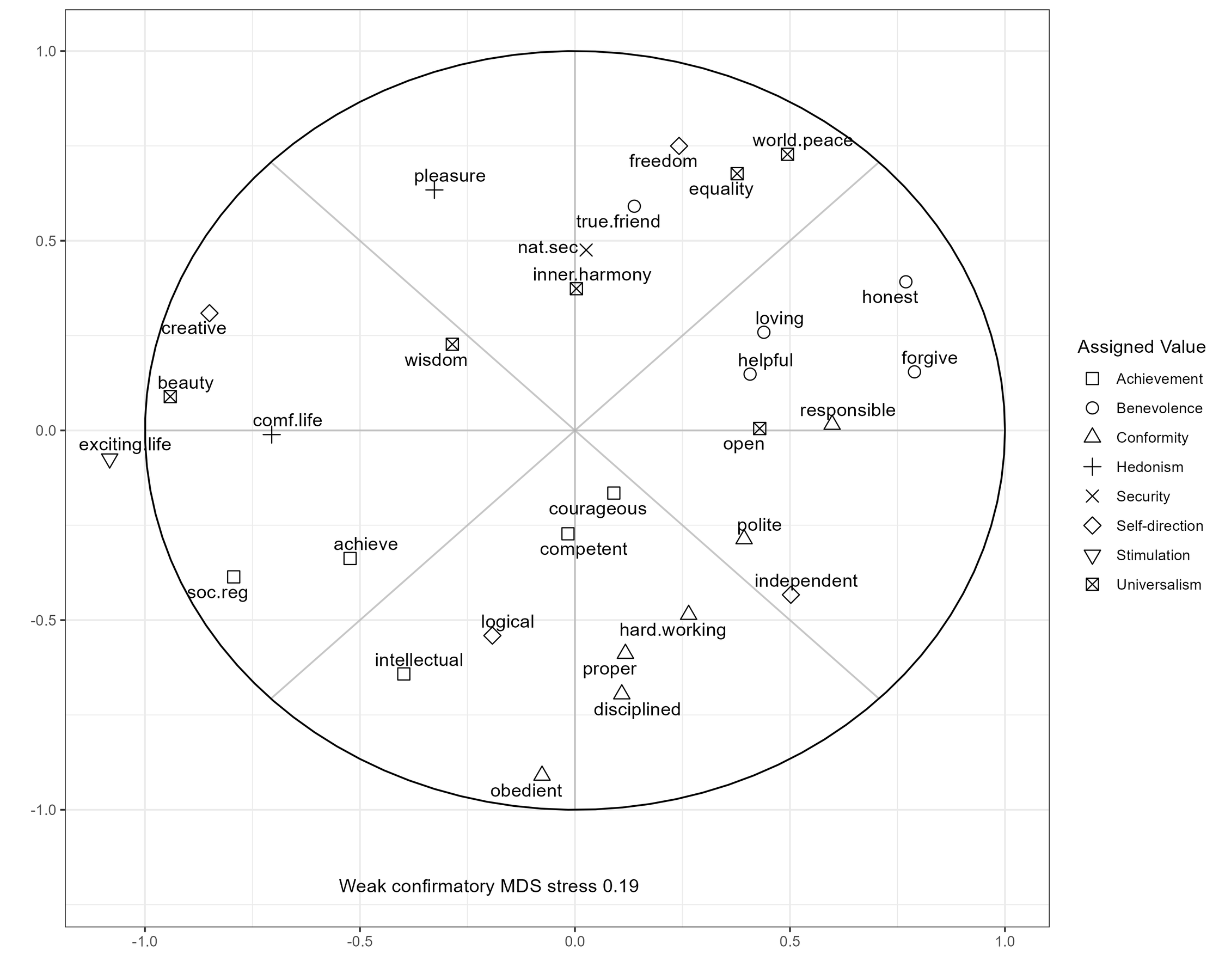

Supplement: S1 Fig — MDS Projections, Heatmaps, and Procrustes Rotation Figures. (ZIP) [file pone.0329179.s001.zip › Renamed Files/Fig21.tif]

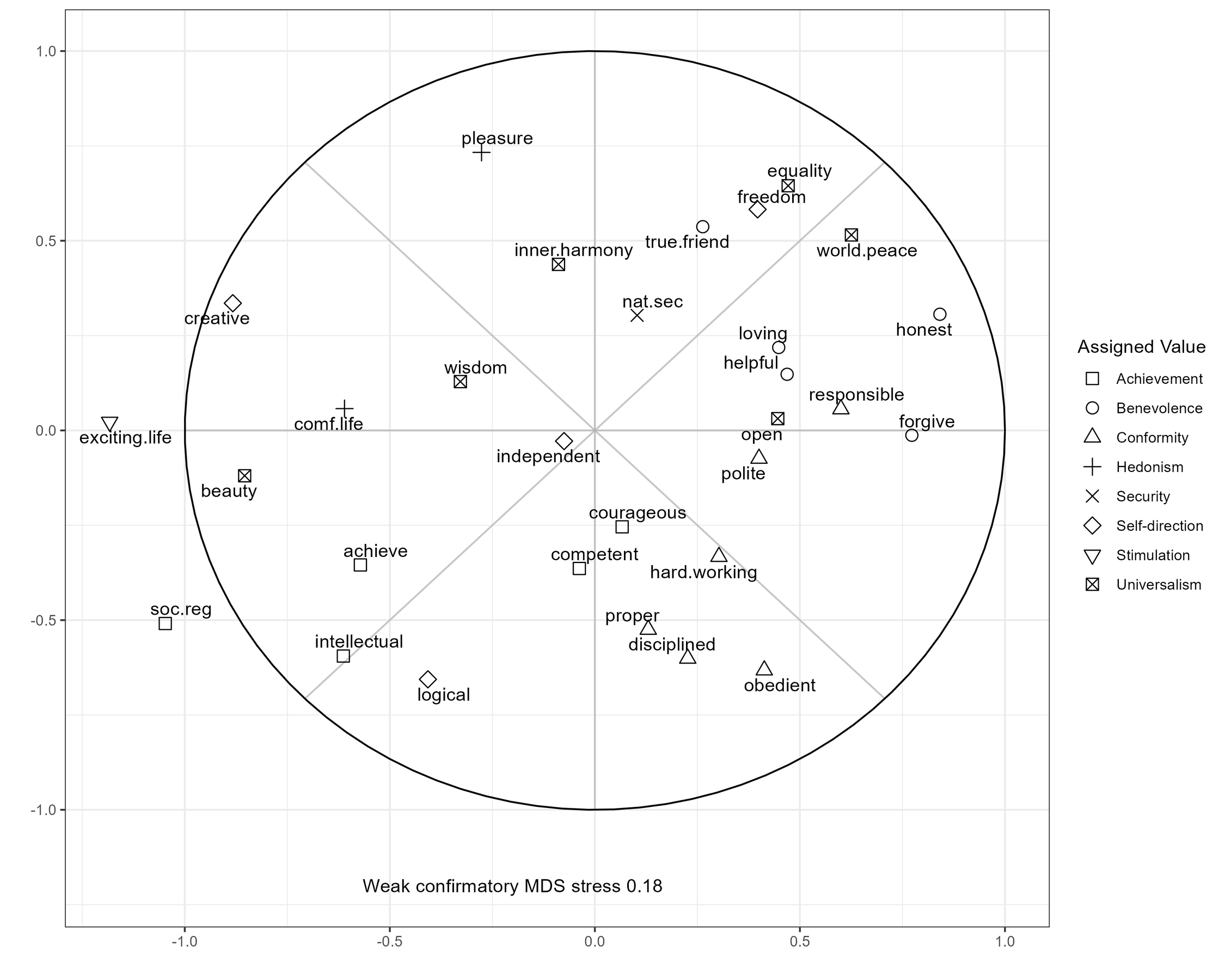

Supplement: S1 Fig — MDS Projections, Heatmaps, and Procrustes Rotation Figures. (ZIP) [file pone.0329179.s001.zip › Renamed Files/Fig22.tif]

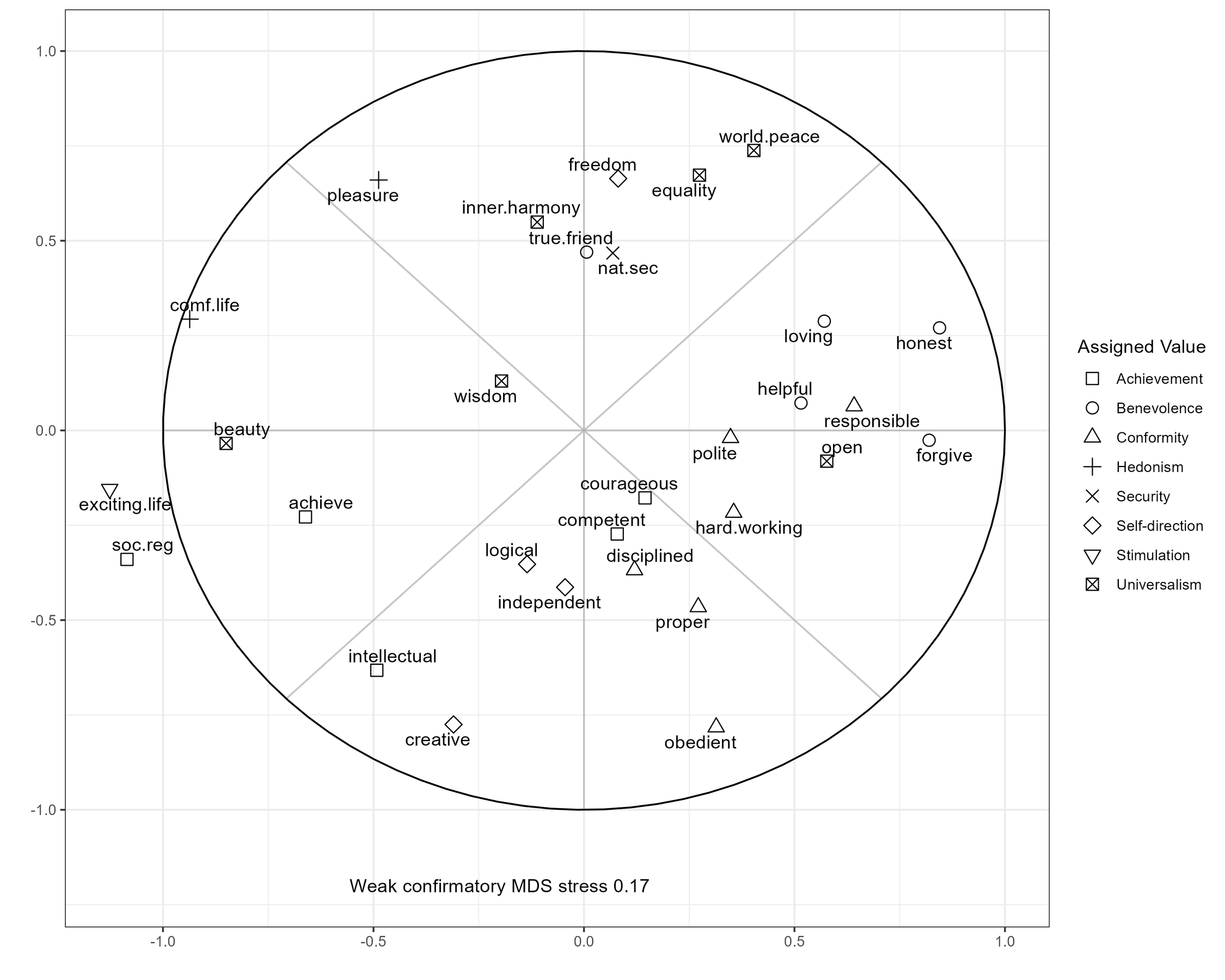

Supplement: S1 Fig — MDS Projections, Heatmaps, and Procrustes Rotation Figures. (ZIP) [file pone.0329179.s001.zip › Renamed Files/Fig29.tif]

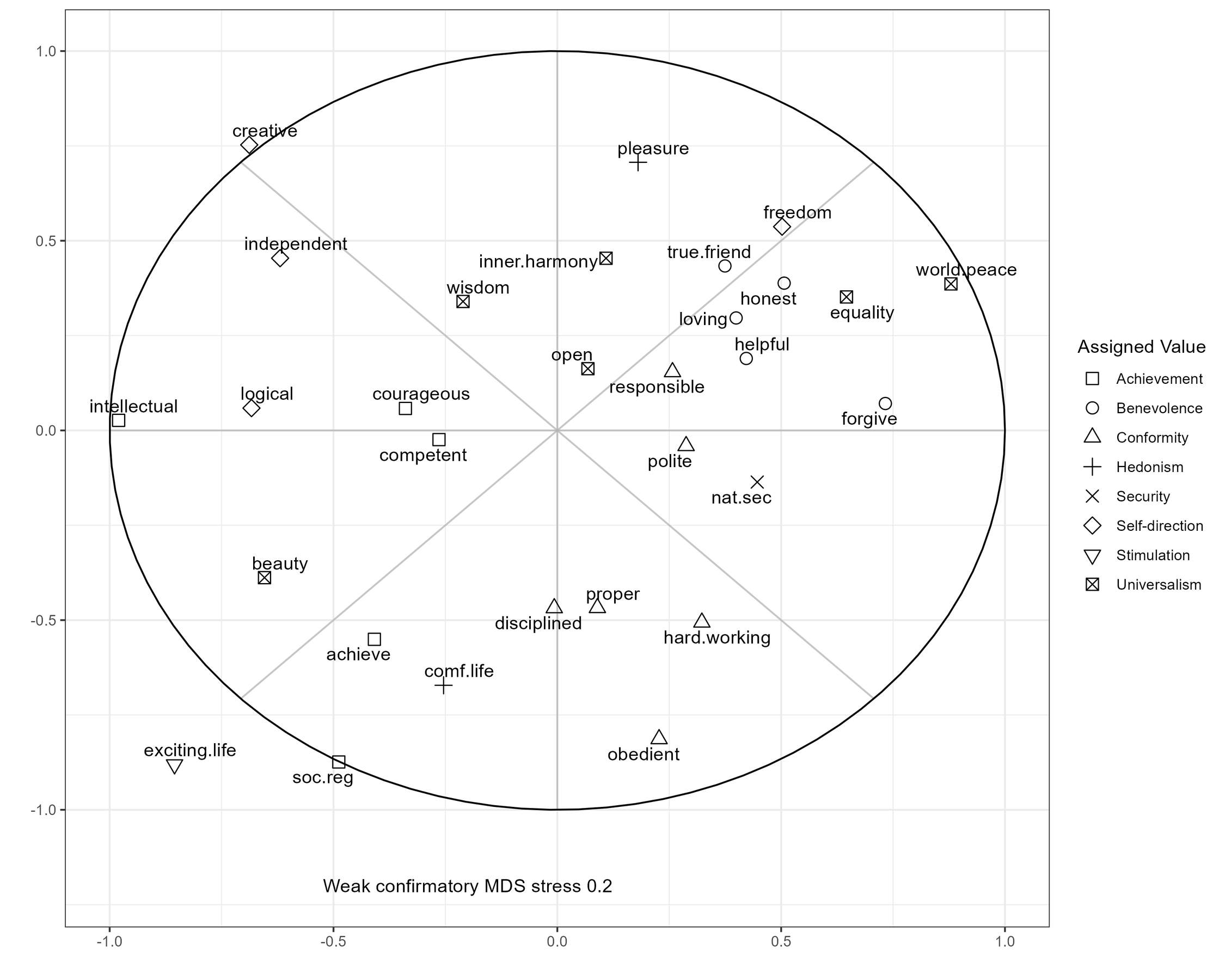

Supplement: S1 Fig — MDS Projections, Heatmaps, and Procrustes Rotation Figures. (ZIP) [file pone.0329179.s001.zip › Renamed Files/Fig30.tif]

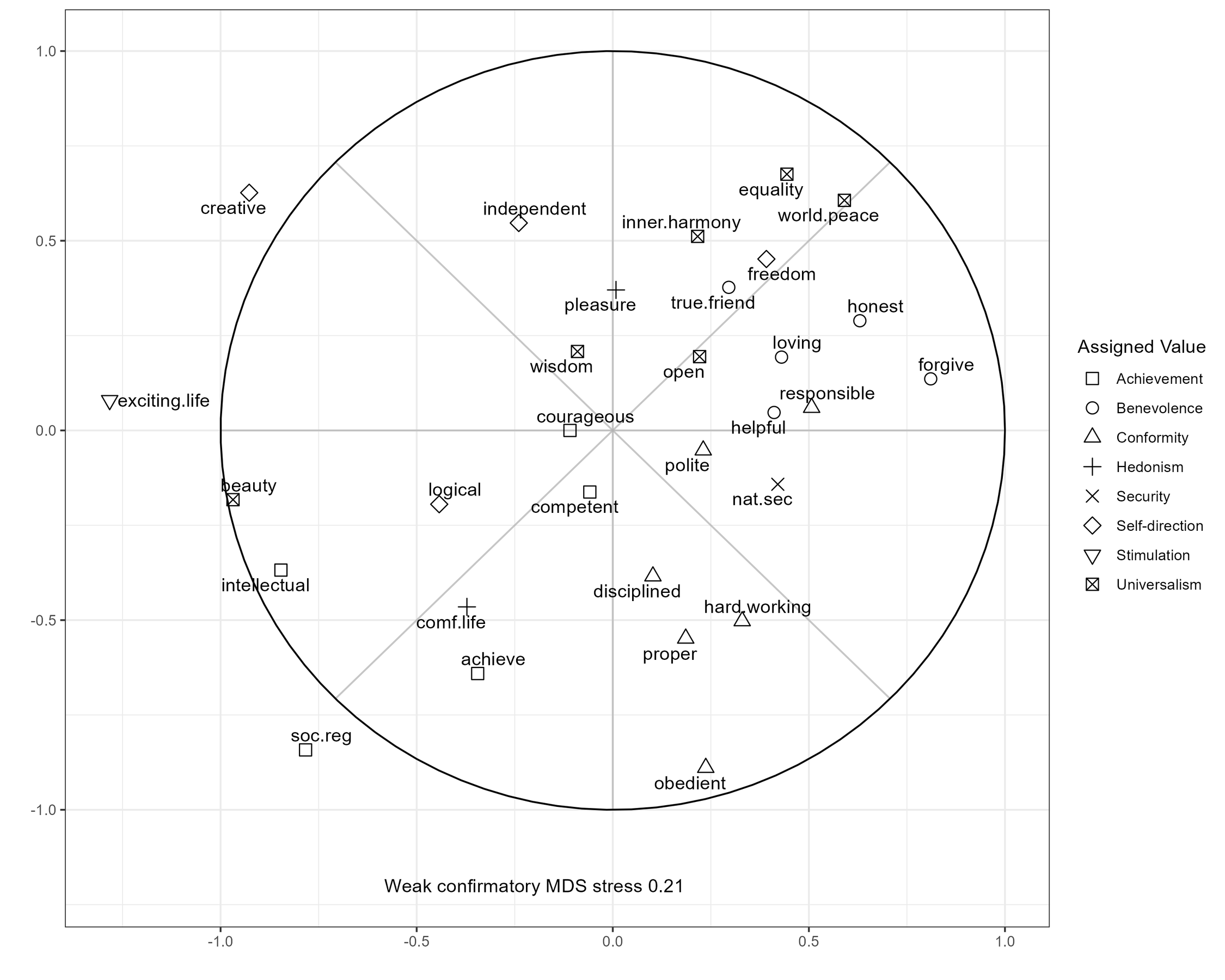

Supplement: S1 Fig — MDS Projections, Heatmaps, and Procrustes Rotation Figures. (ZIP) [file pone.0329179.s001.zip › Renamed Files/Fig27.tif]

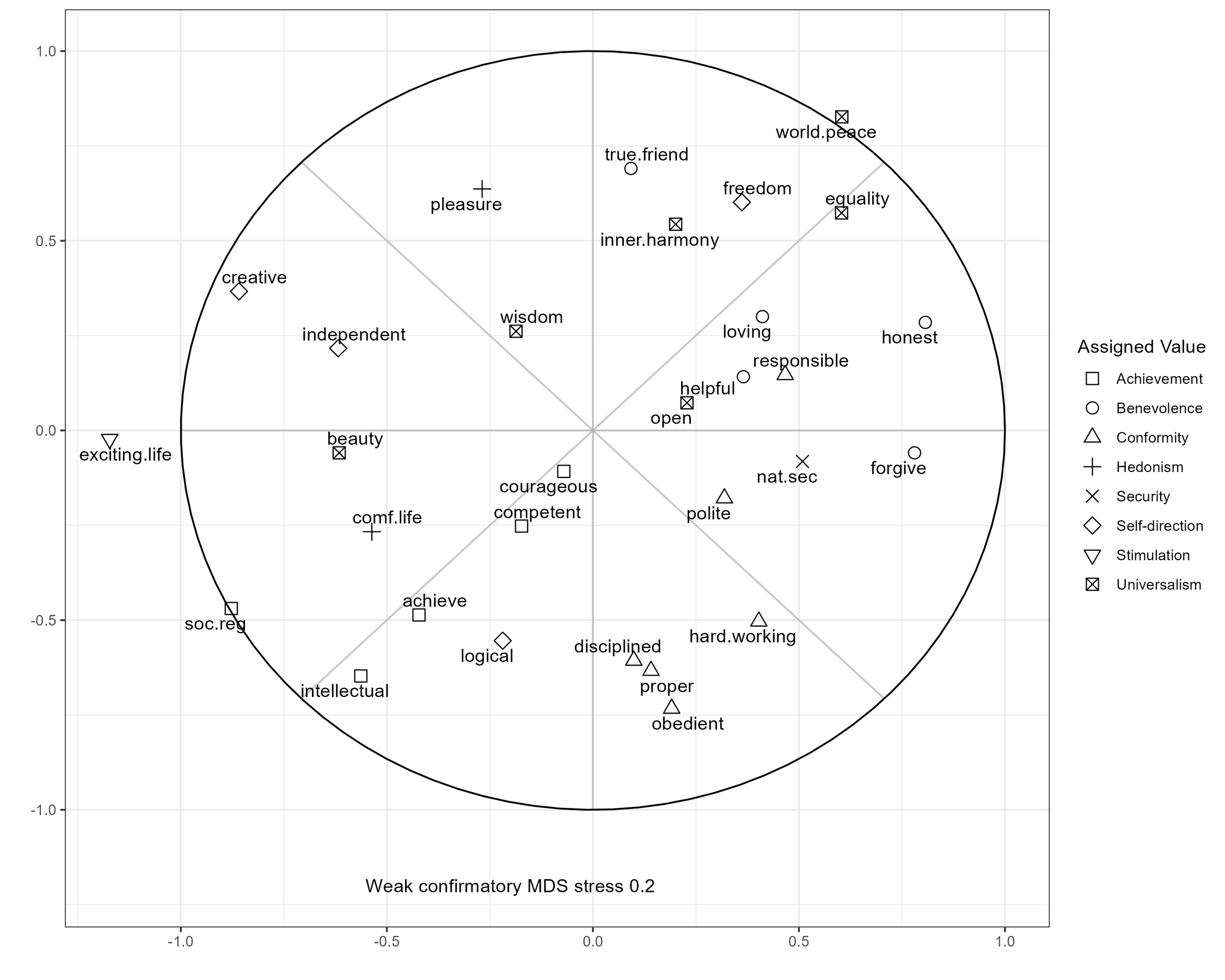

Supplement: S1 Fig — MDS Projections, Heatmaps, and Procrustes Rotation Figures. (ZIP) [file pone.0329179.s001.zip › Renamed Files/Fig28.tif]

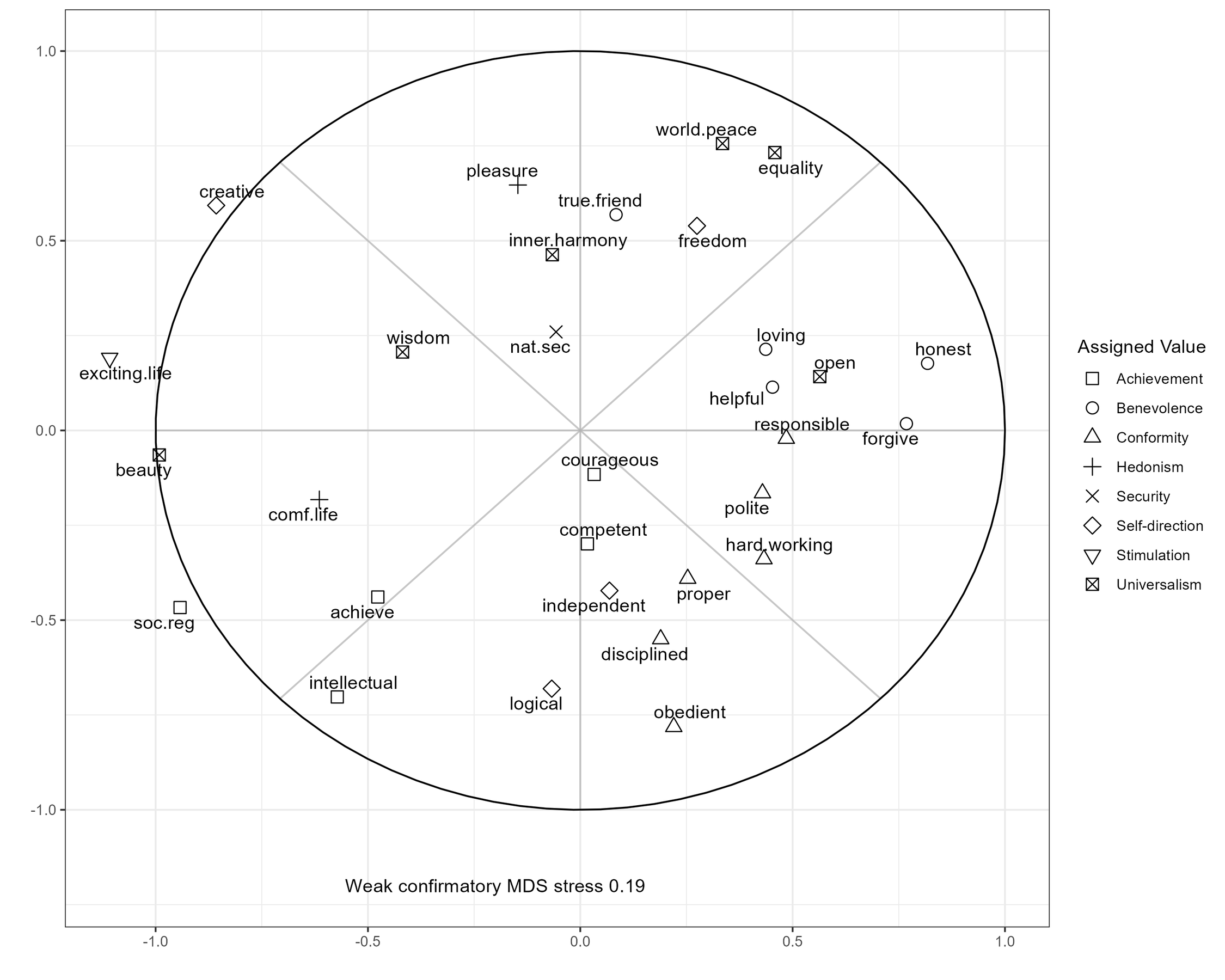

Supplement: S1 Fig — MDS Projections, Heatmaps, and Procrustes Rotation Figures. (ZIP) [file pone.0329179.s001.zip › Renamed Files/Fig25.tif]

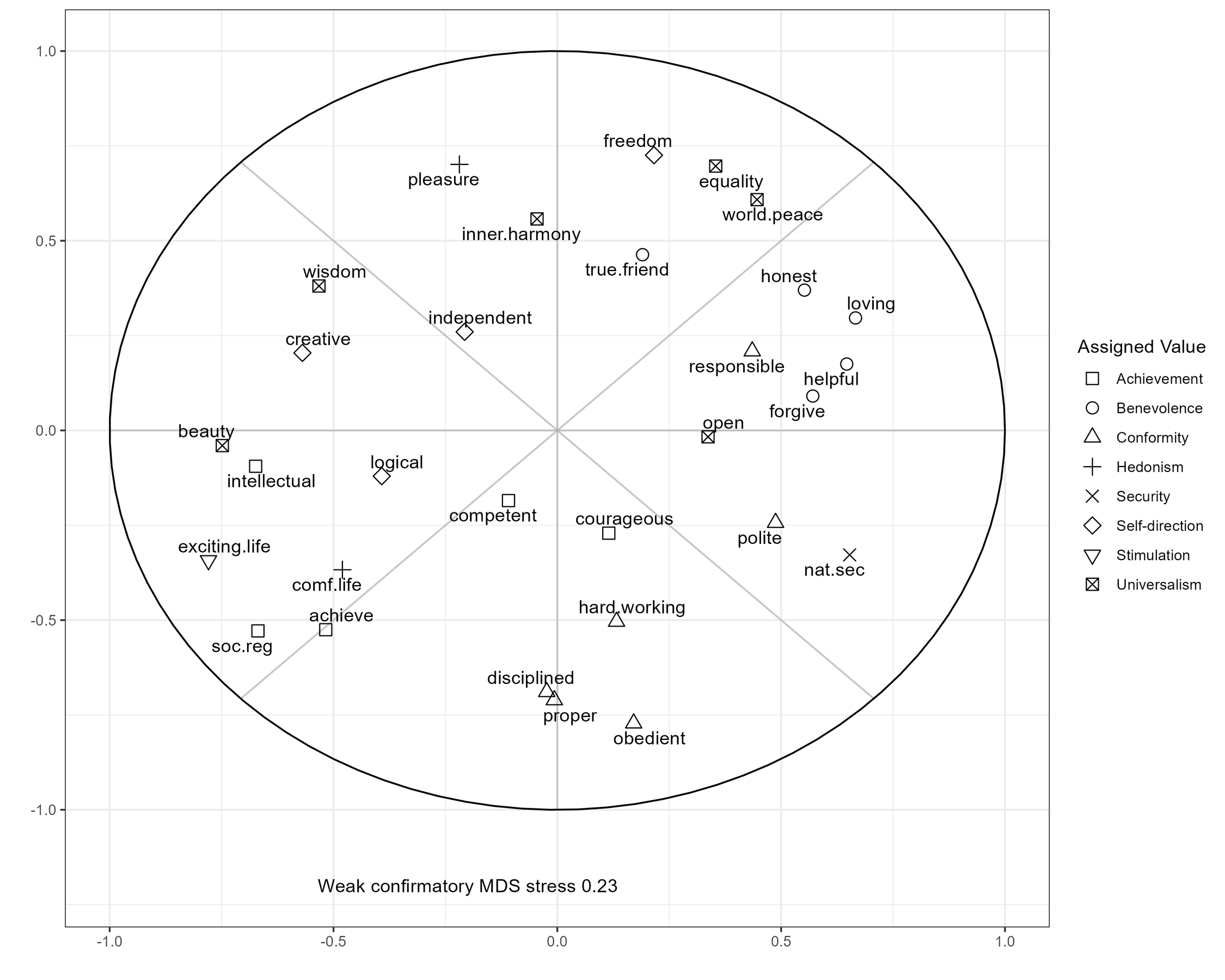

Supplement: S1 Fig — MDS Projections, Heatmaps, and Procrustes Rotation Figures. (ZIP) [file pone.0329179.s001.zip › Renamed Files/Fig5.tif]

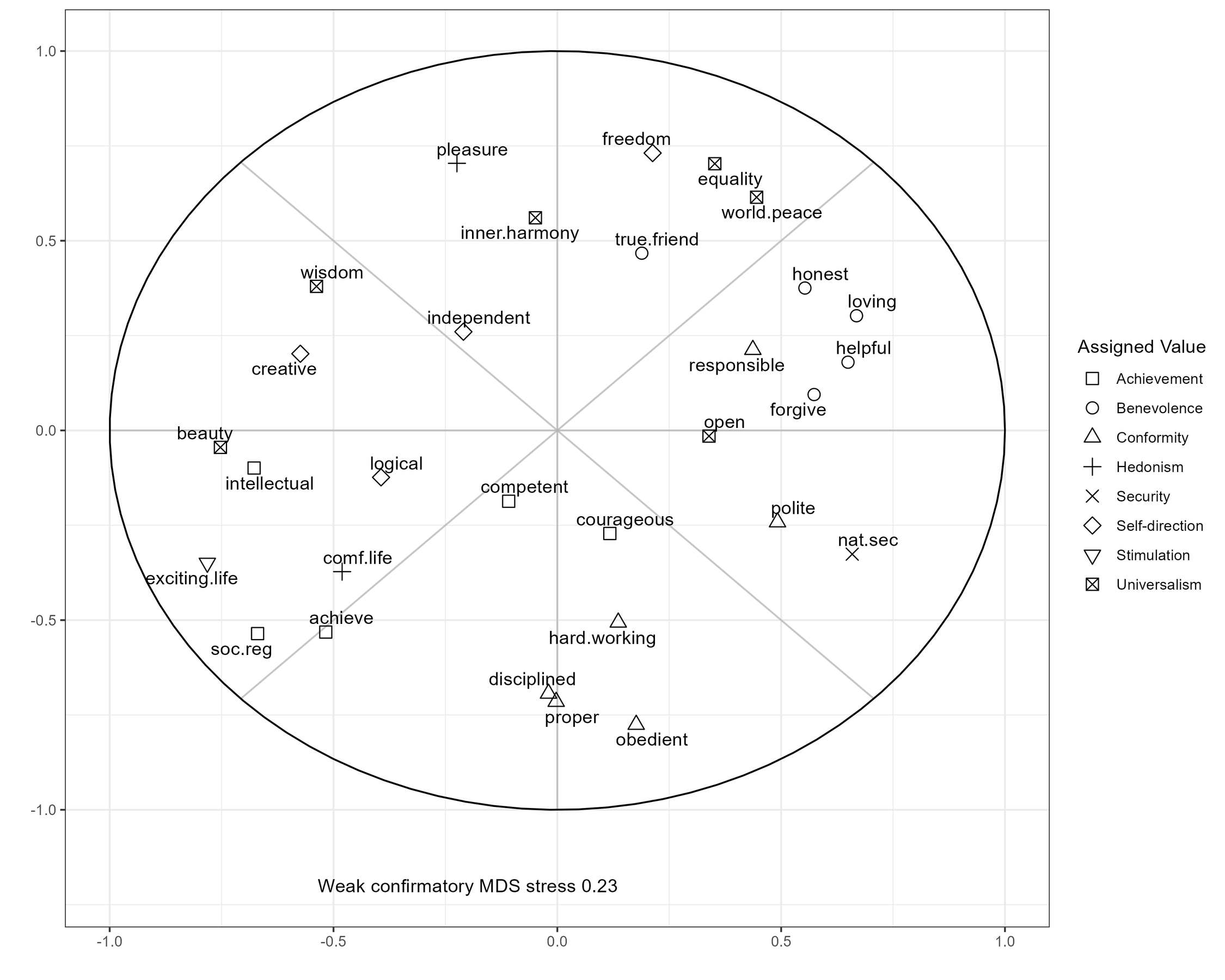

Supplement: S1 Fig — MDS Projections, Heatmaps, and Procrustes Rotation Figures. (ZIP) [file pone.0329179.s001.zip › Renamed Files/Fig6.tif]

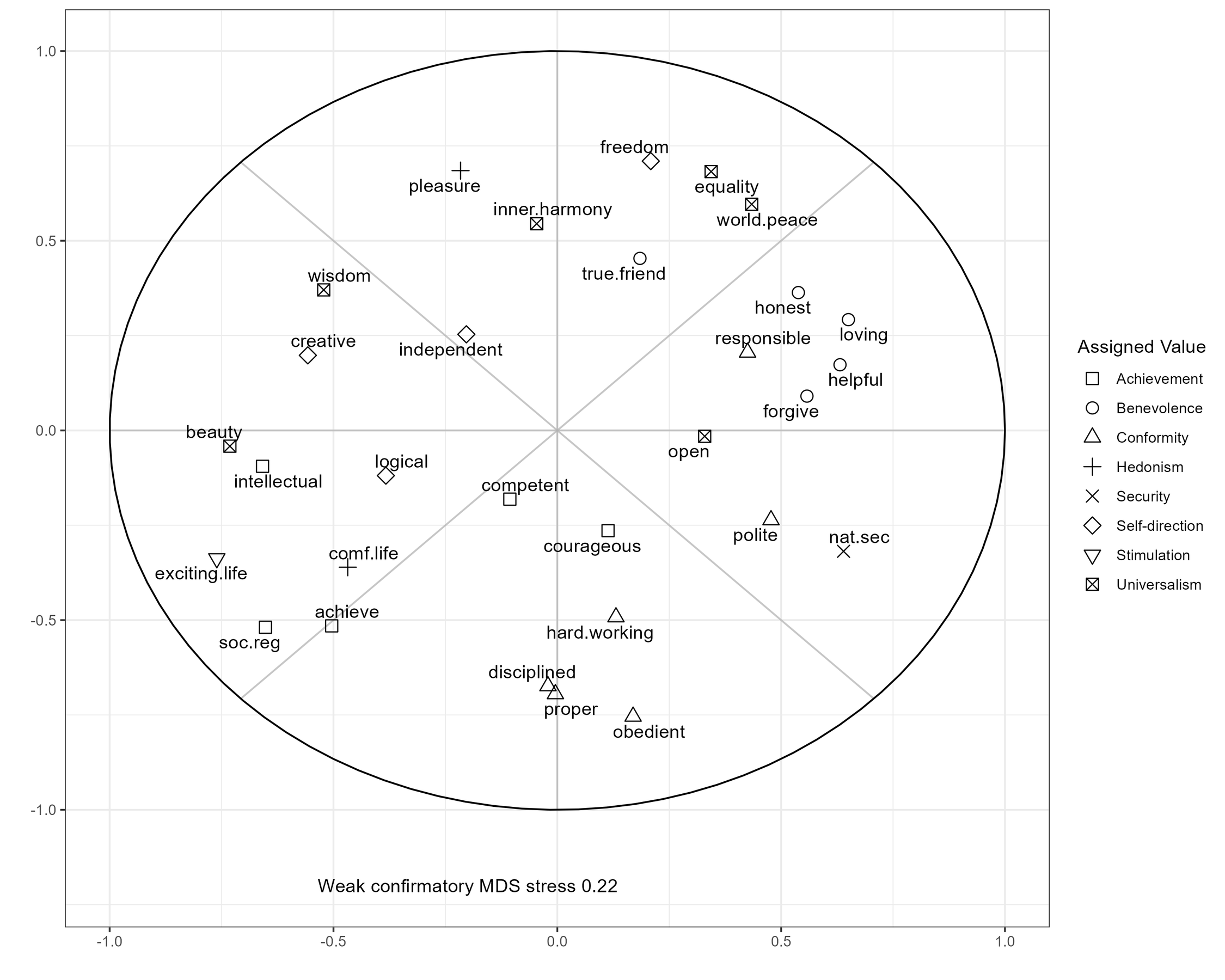

Supplement: S1 Fig — MDS Projections, Heatmaps, and Procrustes Rotation Figures. (ZIP) [file pone.0329179.s001.zip › Renamed Files/Fig7.tif]

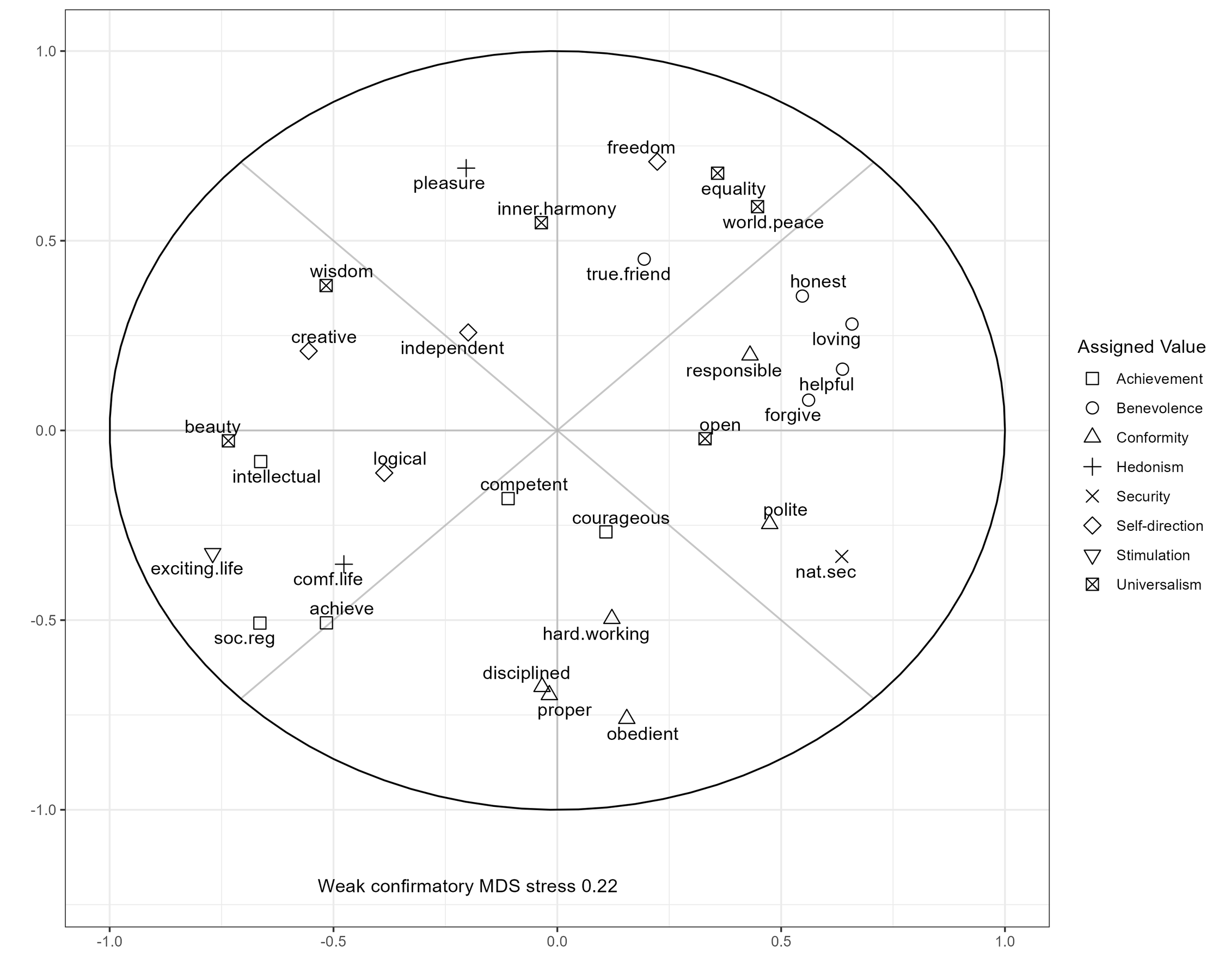

Supplement: S1 Fig — MDS Projections, Heatmaps, and Procrustes Rotation Figures. (ZIP) [file pone.0329179.s001.zip › Renamed Files/Fig8.tif]

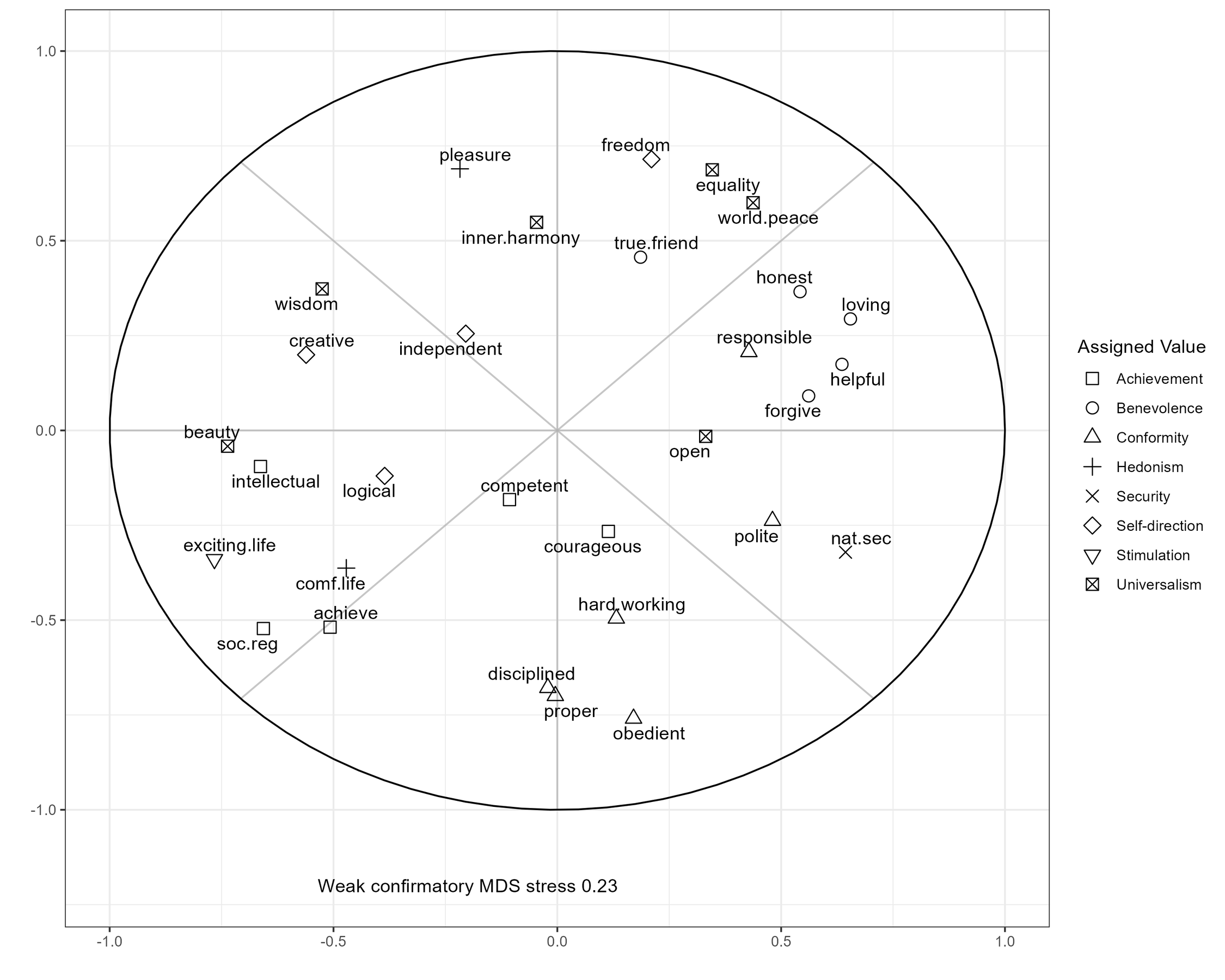

Supplement: S1 Fig — MDS Projections, Heatmaps, and Procrustes Rotation Figures. (ZIP) [file pone.0329179.s001.zip › Renamed Files/Fig9.tif]

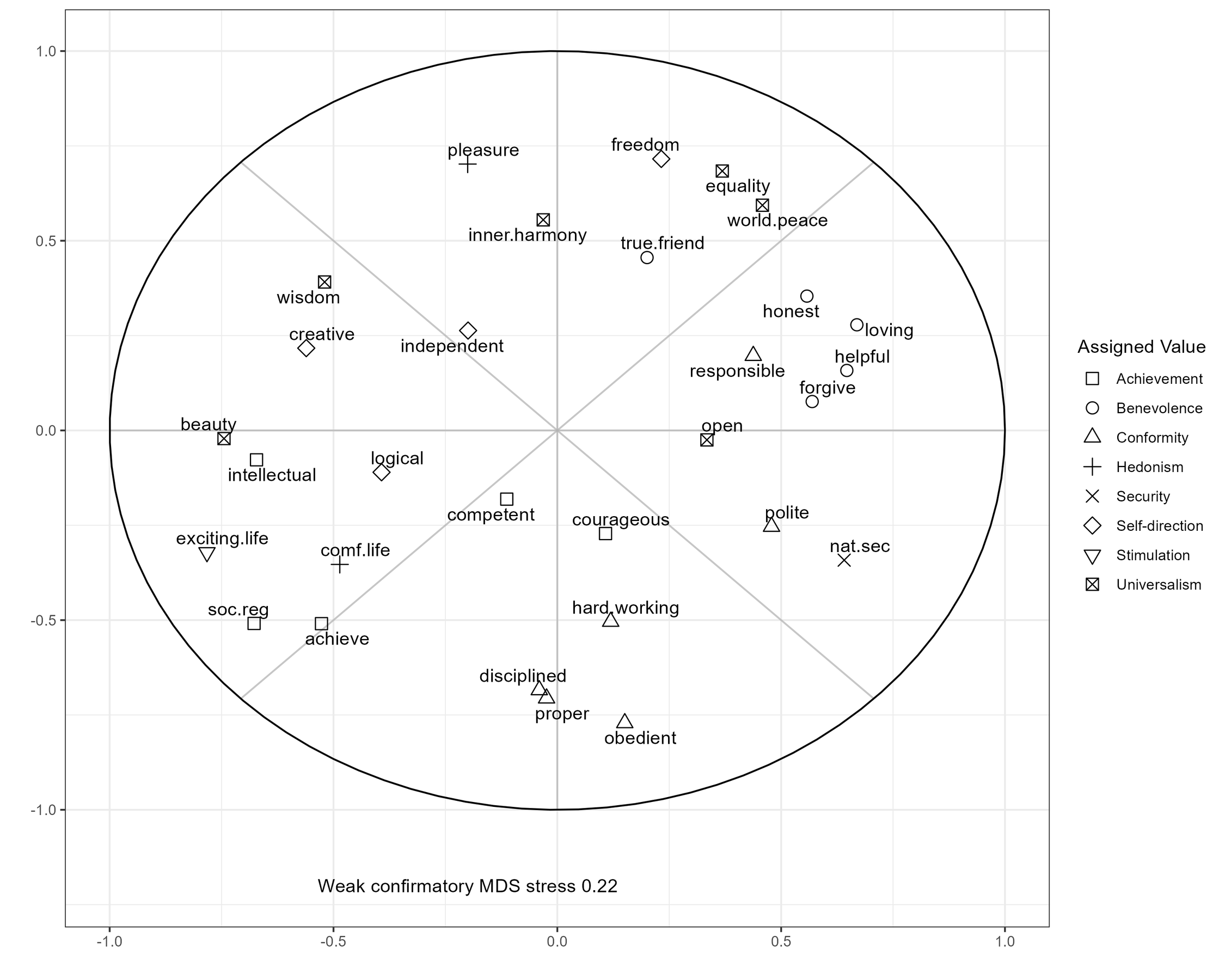

Supplement: S1 Fig — MDS Projections, Heatmaps, and Procrustes Rotation Figures. (ZIP) [file pone.0329179.s001.zip › Renamed Files/Fig10.tif]

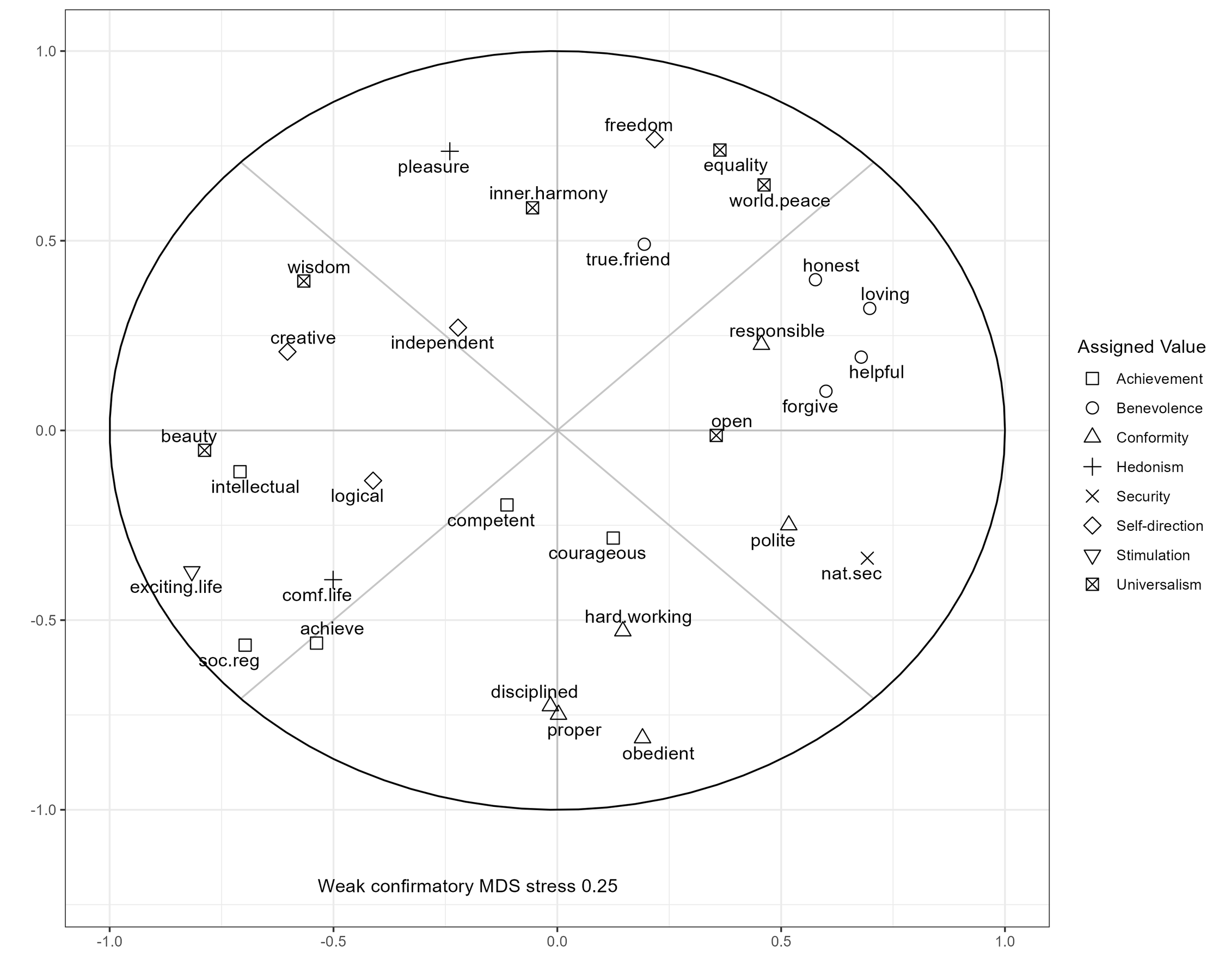

Supplement: S1 Fig — MDS Projections, Heatmaps, and Procrustes Rotation Figures. (ZIP) [file pone.0329179.s001.zip › Renamed Files/Fig1.tif]

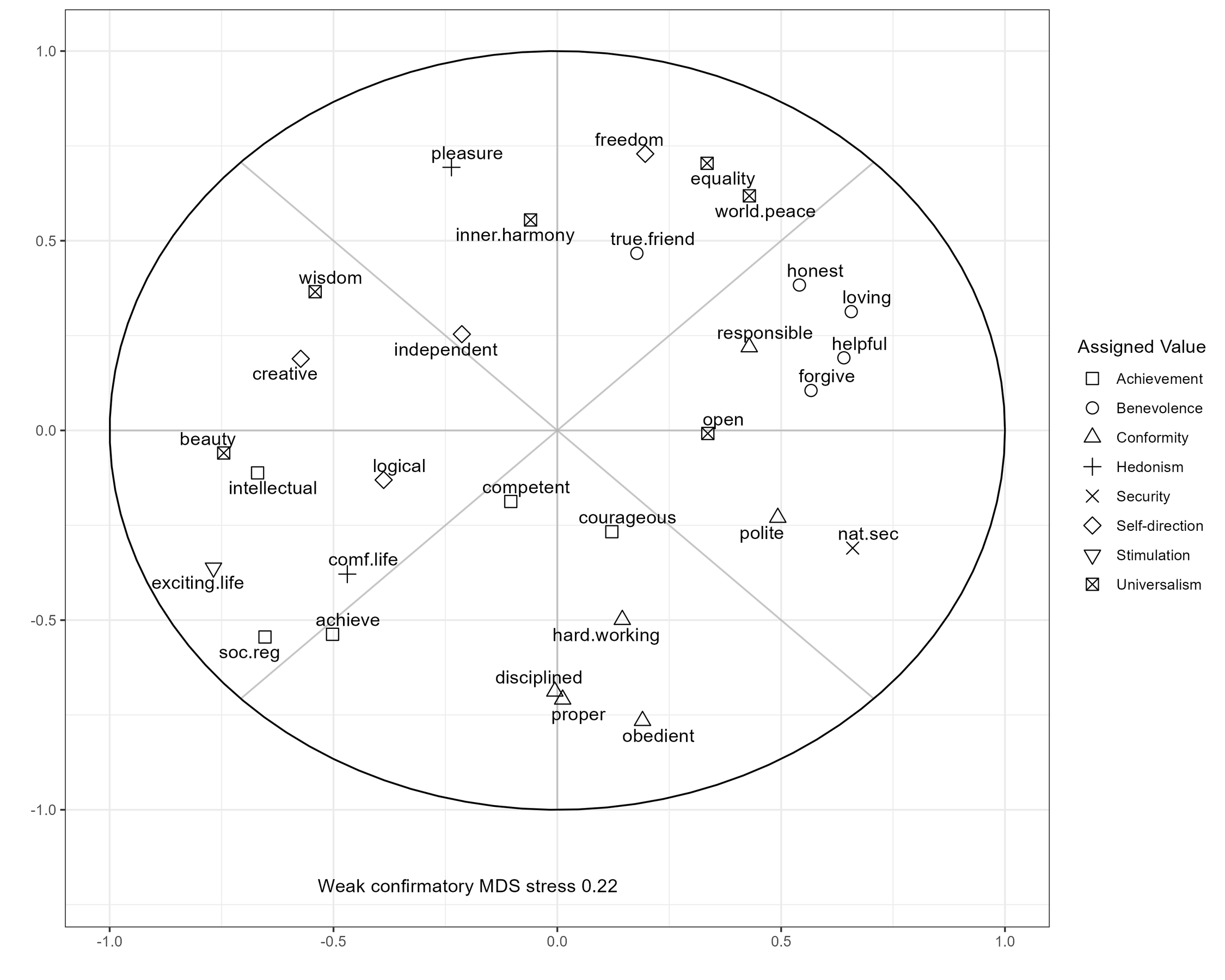

Supplement: S1 Fig — MDS Projections, Heatmaps, and Procrustes Rotation Figures. (ZIP) [file pone.0329179.s001.zip › Renamed Files/Fig2.tif]

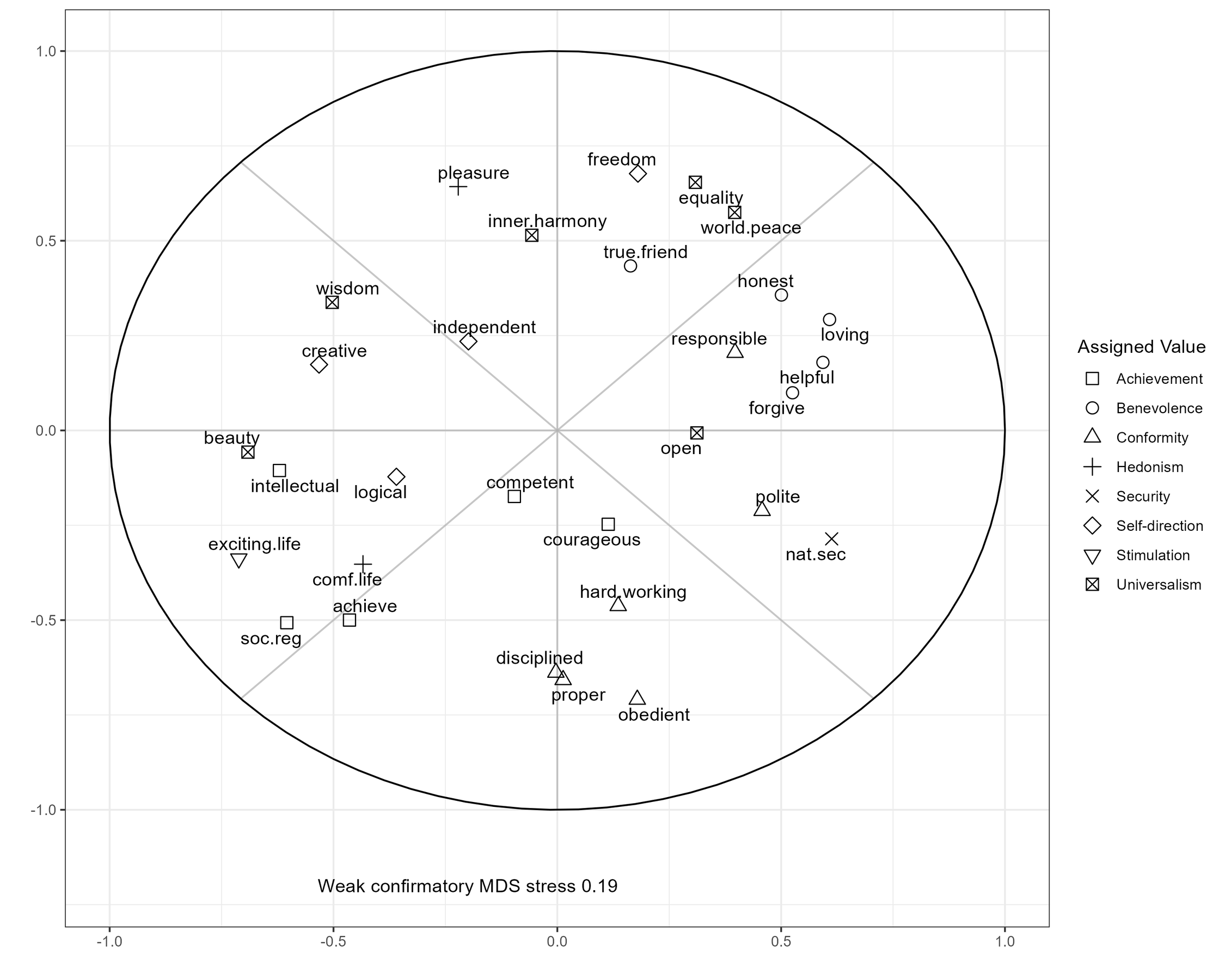

Supplement: S1 Fig — MDS Projections, Heatmaps, and Procrustes Rotation Figures. (ZIP) [file pone.0329179.s001.zip › Renamed Files/Fig3.tif]

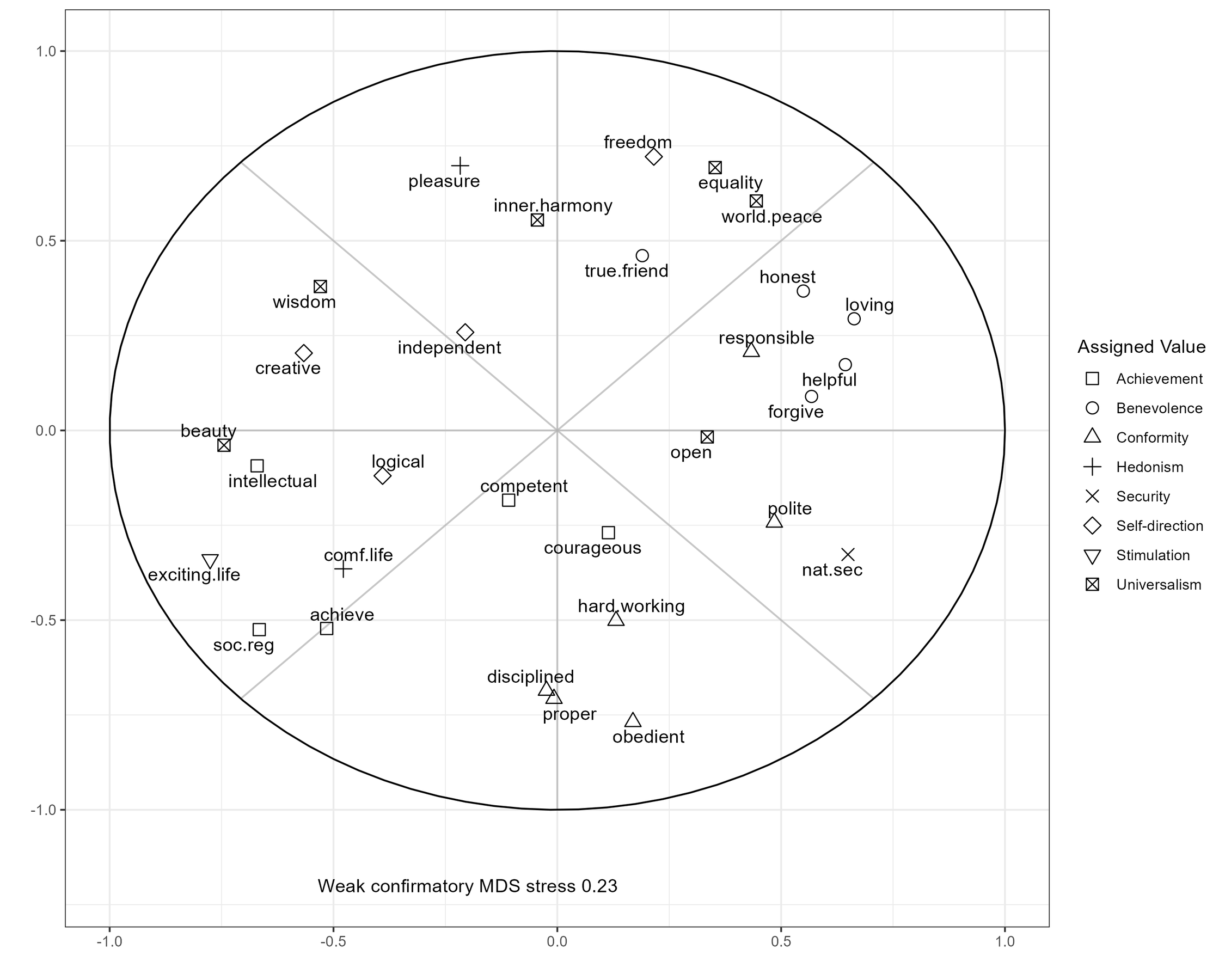

Supplement: S1 Fig — MDS Projections, Heatmaps, and Procrustes Rotation Figures. (ZIP) [file pone.0329179.s001.zip › Renamed Files/Fig4.tif]

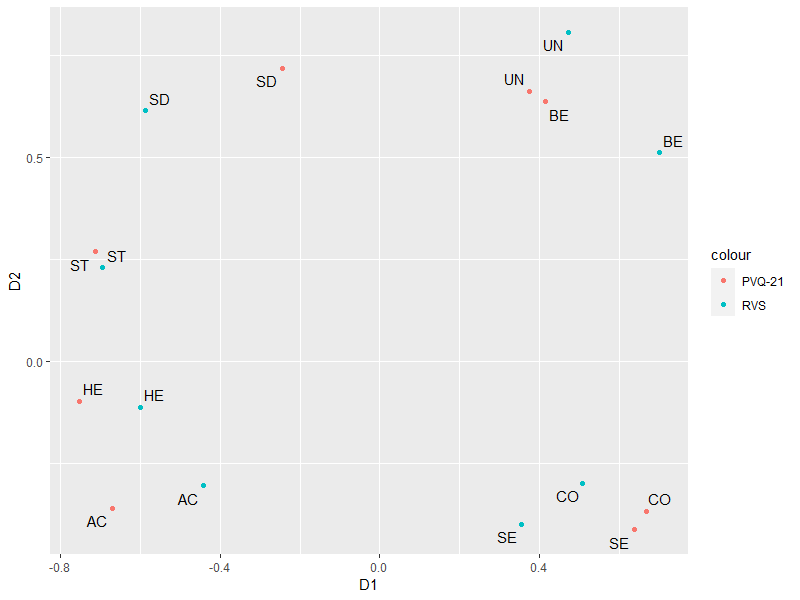

Supplement: S1 Fig — MDS Projections, Heatmaps, and Procrustes Rotation Figures. (ZIP) [file pone.0329179.s001.zip › Renamed Files/Fig32.tif]

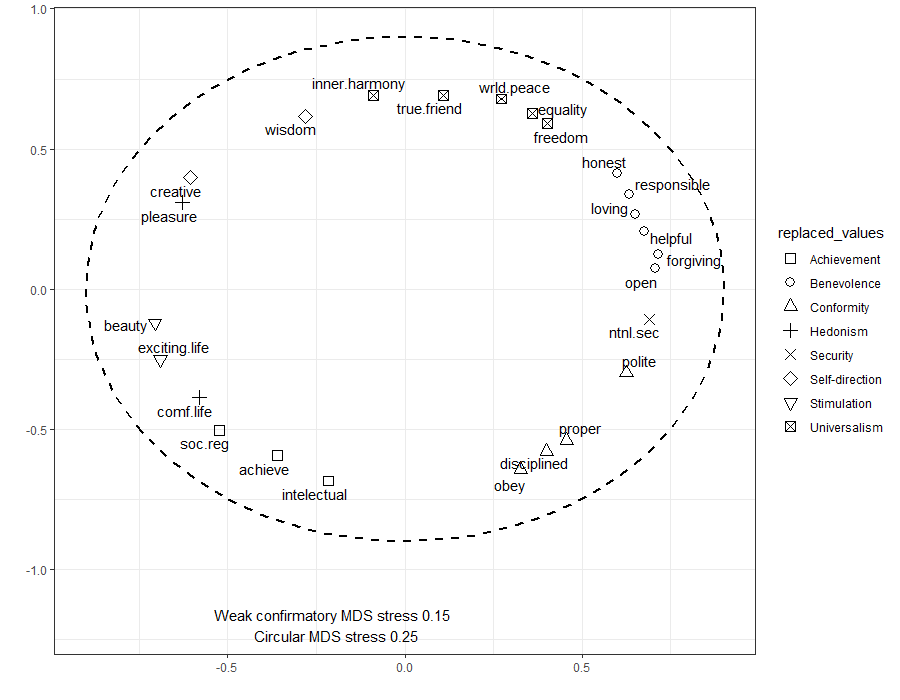

Supplement: S1 Fig — MDS Projections, Heatmaps, and Procrustes Rotation Figures. (ZIP) [file pone.0329179.s001.zip › Renamed Files/Fig31.tif]
