# Supplementary material for: Constructing Schwartz values framework using the Rokeach values survey: Human value measurement in the longitudinal internet survey for social sciences
Source: PLoS One. 2025 Aug 12;20(8):e0329179. doi: 10.1371/journal.pone.0329179 (PMC12342246; doi:10.1371/journal.pone.0329179)
Supplement: S2 Tables — Stress Per Item, Procrustes Rotation Congruence Coefficient, Value Rankings, Regression Tables, Correlation Matrix with Attitudinal Questions, Model Fit Table of the Confirmatory Factor Analysis. (DOCX) [file pone.0329179.s002.docx]

**Table S1.**

*Stress per (ipsatized) RVS item in the weak confirmatory MDS of ten subsamples of the LISS 2008 wave.*

| Item label | Mean | Sample | | | | | | | | | |
| --- | --- | --- | --- | --- | --- | --- | --- | --- | --- | --- | --- |
|  |  | 1 | 2 | 3 | 4 | 5 | 6 | 7 | 8 | 9 | 10 |
| nat.sec | 8.43 | 8.43 | 12.27 | 10.80 | 7.82 | 11.12 | 8.65 | 6.07 | 10.72 | 8.34 | 7.52 |
| pleasure | 5.71 | 5.21 | 5.55 | 4.77 | 4.14 | 5.35 | 8.22 | 6.06 | 4.54 | 6.02 | 6.32 |
| obedient | 5.26 | 6.76 | 3.57 | 3.80 | 3.68 | 4.03 | 5.90 | 5.47 | 2.65 | 8.49 | 3.44 |
| creative | 4.87 | 5.87 | 3.26 | 4.10 | 4.63 | 5.45 | 4.09 | 4.08 | 3.36 | 3.41 | 2.96 |
| courageous | 4.68 | 6.18 | 5.18 | 4.07 | 4.48 | 4.83 | 6.04 | 5.79 | 5.61 | 3.79 | 2.99 |
| comf.life | 4.29 | 2.79 | 4.33 | 3.45 | 7.47 | 5.30 | 4.49 | 4.29 | 6.43 | 2.92 | 6.35 |
| independent | 4.24 | 4.74 | 4.24 | 4.58 | 4.17 | 5.08 | 5.55 | 4.81 | 4.47 | 4.58 | 3.06 |
| polite | 4.24 | 5.74 | 3.52 | 3.42 | 4.42 | 4.66 | 4.05 | 5.79 | 3.56 | 3.67 | 2.72 |
| wisdom | 3.82 | 4.32 | 2.43 | 4.38 | 4.56 | 3.03 | 3.50 | 4.81 | 5.51 | 5.19 | 3.28 |
| exciting.life | 3.45 | 2.45 | 2.10 | 2.29 | 2.76 | 2.07 | 1.96 | 3.03 | 2.87 | 2.73 | 4.96 |
| hard.working | 3.34 | 3.84 | 4.61 | 3.97 | 3.00 | 3.92 | 3.17 | 4.12 | 2.65 | 4.69 | 2.78 |
| world.peace | 3.25 | 1.75 | 3.81 | 4.94 | 4.74 | 3.32 | 4.20 | 2.75 | 4.23 | 2.33 | 5.32 |
| competent | 3.19 | 3.19 | 4.31 | 3.16 | 2.78 | 3.06 | 3.09 | 3.68 | 3.00 | 3.02 | 3.46 |
| soc.reg | 3.15 | 3.15 | 2.52 | 2.07 | 3.13 | 2.21 | 1.42 | 1.59 | 1.85 | 2.50 | 2.82 |
| responsible | 2.96 | 2.96 | 2.85 | 3.44 | 3.37 | 2.30 | 2.11 | 3.14 | 2.12 | 1.50 | 2.91 |
| open | 2.91 | 2.41 | 3.86 | 3.96 | 5.75 | 2.71 | 4.16 | 3.03 | 4.58 | 3.40 | 3.42 |
| true.friend | 2.85 | 2.35 | 2.33 | 4.72 | 2.28 | 3.50 | 3.39 | 3.49 | 3.89 | 2.42 | 3.84 |
| proper | 2.76 | 3.76 | 2.01 | 2.03 | 0.95 | 2.60 | 3.54 | 3.90 | 1.64 | 4.58 | 1.55 |
| logical | 2.72 | 2.22 | 1.98 | 1.33 | 1.33 | 2.40 | 2.09 | 1.38 | 3.31 | 2.85 | 3.55 |
| disciplined | 2.35 | 2.85 | 1.59 | 1.71 | 1.49 | 1.58 | 2.23 | 1.86 | 0.84 | 2.14 | 1.16 |
| inner.harmony | 2.35 | 2.35 | 2.30 | 1.83 | 2.30 | 2.91 | 2.30 | 1.95 | 3.14 | 2.19 | 2.47 |
| beauty | 2.31 | 2.31 | 3.39 | 3.54 | 2.98 | 2.71 | 1.71 | 1.93 | 1.64 | 3.13 | 2.58 |
| loving | 2.28 | 1.78 | 2.81 | 2.08 | 1.78 | 1.39 | 1.48 | 2.24 | 1.96 | 2.93 | 3.35 |
| honest | 2.23 | 2.23 | 2.10 | 2.55 | 2.12 | 2.06 | 2.30 | 2.00 | 2.37 | 2.63 | 1.96 |
| intellectual | 2.18 | 2.18 | 1.51 | 1.09 | 1.19 | 2.20 | 2.42 | 1.82 | 2.14 | 1.43 | 2.75 |
| achieve | 2.18 | 2.18 | 2.54 | 1.65 | 2.68 | 2.72 | 1.97 | 2.73 | 1.76 | 1.94 | 2.59 |
| equality | 1.84 | 1.34 | 2.48 | 2.21 | 2.23 | 2.34 | 1.01 | 1.54 | 2.38 | 1.59 | 2.92 |
| forgive | 1.79 | 1.29 | 2.67 | 1.97 | 2.66 | 1.97 | 2.22 | 2.23 | 3.10 | 1.44 | 2.63 |
| helpful | 1.73 | 1.73 | 1.75 | 3.22 | 3.04 | 1.48 | 0.95 | 1.57 | 1.47 | 1.12 | 1.83 |
| freedom | 1.65 | 1.65 | 2.13 | 2.86 | 2.07 | 1.71 | 1.78 | 2.84 | 2.20 | 3.04 | 2.53 |

*Note*. items above the dotted line have a mean stress per item higher than 1/30^th^ (3.3%).

**Table S2.**

*Procrustes rotation congruence coefficients of all pairwise combinations of the 10 ipsatized sub-samples of the RVS wave 2008.*

| Sample | Sample | Congruence Coefficient | Sample | Sample | Congruence coefficient |
| --- | --- | --- | --- | --- | --- |
| 1 | 2 | 0.97 | 2 | 10 | 0.97 |
| 1 | 3 | 0.98 | 3 | 4 | 0.99 |
| 1 | 4 | 0.97 | 3 | 5 | 0.98 |
| 1 | 5 | 0.99 | 3 | 6 | 0.98 |
| 1 | 6 | 0.99 | 3 | 7 | 0.98 |
| 1 | 7 | 0.99 | 3 | 8 | 0.99 |
| 1 | 8 | 0.97 | 3 | 9 | 0.98 |
| 1 | 9 | 0.99 | 3 | 10 | 0.97 |
| 1 | 10 | 0.95 | 4 | 5 | 0.96 |
| 2 | 3 | 0.99 | 4 | 6 | 0.96 |
| 2 | 4 | 0.98 | 4 | 7 | 0.97 |
| 2 | 5 | 0.97 | 4 | 8 | 0.98 |
| 2 | 6 | 0.97 | 4 | 9 | 0.97 |
| 2 | 7 | 0.97 | 4 | 10 | 0.97 |
| 2 | 8 | 0.98 | 5 | 6 | 0.99 |
| 2 | 9 | 0.97 | 5 | 7 | 0.99 |
| 7 | 8 | 0.97 | 5 | 8 | 0.97 |
| 7 | 9 | 0.99 | 5 | 9 | 0.98 |
| 7 | 10 | 0.95 | 5 | 10 | 0.96 |
| 8 | 9 | 0.96 | 6 | 7 | 0.99 |
| 8 | 10 | 0.99 | 6 | 8 | 0.97 |
| 9 | 10 | 0.95 | 6 | 9 | 0.99 |
|  |  |  | 6 | 10 | 0.96 |

**Table S3.**

*Rankings of values by scale all ages*

| Variable | RVS 2013-US-Revised | WVS 2012-PVQ-11 | ESS 2012 PVQ-21 |
| --- | --- | --- | --- |
| BE | 1 | 1 | 1 |
| SD | 5 | 5 | 2 |
| UN | 2 | 2 | 3 |
| SE | 3 | 3 | 4 |
| HE | 4 | 6 | 5 |
| CO | 6 | 4 | 6 |
| TR | NA | 7 | 7 |
| AC | 7 | 8 | 8 |
| ST | 8 | 9 | 9 |
| PO | NA | 10 | 10 |

**Table S4.**

*Rankings of values by scale ages 25-35*

| Variable | RVS 2013-US-Revised | WVS 2012-PVQ-11 | ESS 2012 PVQ-21 |
| --- | --- | --- | --- |
| BE | 1 | 2 | 1 |
| SD | 6 | 4 | 2 |
| UN | 3 | 6 | 3 |
| HE | 2 | 1 | 4 |
| AC | 7 | 7 | 5 |
| SE | 4 | 5 | 6 |
| ST | 8 | 9 | 7 |
| CO | 5 | 3 | 8 |
| TR | NA | 8 | 9 |
| PO | NA | 10 | 10 |

**Table S5.**

*Rankings of values by scale ages 36-45*

| Variable | RVS 2013-US-Revised | WVS 2012-PVQ-11 | ESS 2012 PVQ-21 |
| --- | --- | --- | --- |
| BE | 1 | 1 | 1 |
| SD | 6 | 6 | 2 |
| UN | 2 | 5 | 3 |
| HE | 3 | 2 | 4 |
| SE | 4 | 3 | 5 |
| AC | 8 | 8 | 6 |
| CO | 5 | 4 | 7 |
| ST | 7 | 9 | 8 |
| TR | NA | 7 | 9 |
| PO | NA | 10 | 10 |

**Table S6.**

*Rankings of values by scale ages 46-56*

| Variable | RVS 2013-US-Revised | WVS 2012-PVQ-11 | ESS 2012 PVQ-21 |
| --- | --- | --- | --- |
| BE | 1 | 1 | 1 |
| UN | 2 | 2 | 2 |
| SD | 5 | 6 | 3 |
| SE | 3 | 3 | 4 |
| HE | 4 | 5 | 5 |
| CO | 6 | 4 | 6 |
| TR | NA | 7 | 7 |
| ST | 8 | 8 | 8 |
| AC | 7 | 9 | 9 |
| PO | NA | 10 | 10 |

**Table S7.**

*Rankings of values by scale ages 57-67*

| Variable | RVS 2013-US-Revised | WVS 2012-PVQ-11 | ESS 2012 PVQ-21 |
| --- | --- | --- | --- |
| BE | 2 | 1 | 1 |
| UN | 1 | 2 | 2 |
| SD | 5 | 5 | 3 |
| SE | 3 | 3 | 4 |
| CO | 6 | 4 | 5 |
| HE | 4 | 6 | 6 |
| TR | NA | 7 | 7 |
| ST | 8 | 8 | 8 |
| AC | 7 | 9 | 9 |
| PO | NA | 10 | 10 |

**Table S8.**

*Descriptive statistics of RVS-US-Revised value types in Wave 2013*

| Variable | mean | sd | min | max | Ranking |
| --- | --- | --- | --- | --- | --- |
| BE | 0.61 | 0.61 | -3.88 | 3.08 | 1 |
| UN | 0.54 | 0.59 | -2.13 | 2.81 | 2 |
| SE | 0.31 | 0.94 | -3.97 | 3.21 | 3 |
| HE | 0.26 | 0.63 | -2.44 | 2.86 | 4 |
| SD | -0.10 | 0.72 | -3.39 | 2.97 | 5 |
| CO | -0.13 | 0.70 | -3.88 | 2.23 | 6 |
| AC | -0.71 | 0.72 | -4.08 | 2.18 | 7 |
| ST | -0.79 | 0.85 | -4.62 | 2.67 | 8 |

**Table S9.**

*Descriptive statistics of ESS PVQ-21 value types in Wave 2012*

| Variable | mean | sd | min | max | Ranking |
| --- | --- | --- | --- | --- | --- |
| BE | 0.68 | 0.58 | -1.48 | 2.60 | 1 |
| SD | 0.61 | 0.67 | -2.45 | 2.52 | 2 |
| UN | 0.57 | 0.58 | -1.81 | 2.52 | 3 |
| SE | 0.04 | 0.77 | -3.38 | 3.81 | 4 |
| HE | 0.03 | 0.71 | -3.07 | 2.38 | 5 |
| CO | -0.18 | 0.84 | -3.33 | 2.14 | 6 |
| TR | -0.31 | 0.86 | -3.52 | 2.38 | 7 |
| AC | -0.35 | 0.80 | -3.57 | 2.62 | 8 |
| ST | -0.40 | 0.88 | -3.43 | 2.62 | 9 |
| PO | -0.99 | 0.76 | -3.63 | 2.02 | 10 |

**Table S10.**

*Descriptive statistics of WVS PVQ-11 value types in Wave 2012*

| Variable | mean | sd | min | max | Ranking |
| --- | --- | --- | --- | --- | --- |
| BE | 0.83 | 0.98 | -2.64 | 3.55 | 1 |
| UN | 0.56 | 0.75 | -2.00 | 2.62 | 2 |
| SE | 0.39 | 1.11 | -3.36 | 3.00 | 3 |
| CO | 0.35 | 1.10 | -2.73 | 3.00 | 4 |
| SD | 0.28 | 1.17 | -3.09 | 3.27 | 5 |
| HE | 0.27 | 0.99 | -2.82 | 3.10 | 6 |
| TR | -0.32 | 1.19 | -3.27 | 3.45 | 7 |
| AC | -0.83 | 1.00 | -3.45 | 2.82 | 8 |
| ST | -0.86 | 1.12 | -3.64 | 2.91 | 9 |
| PO | -1.21 | 0.92 | -4.09 | 2.64 | 10 |

**Table S11.**

*Regression results of Universalism scales on socio-demographics in the European Social Survey, the Longitudinal internet study for social sciences and the world value survey.*

|  | ESS PVQ-21 | LISS RVS | WVS PVQ-11 |
| --- | --- | --- | --- |
|  | (1) | (2) | (3) |
|  | | | |
| age | 0.19^***^ | 0.21^***^ | 0.32^***^ |
|  | (0.03) | (0.02) | (0.03) |
|  |  |  |  |
| Male | -0.30^***^ | -0.39^***^ | -0.28^***^ |
|  | (0.05) | (0.03) | (0.05) |
|  |  |  |  |
| Primary | -0.31^**^ | -0.04 | 0.02 |
|  | (0.12) | (0.08) | (0.13) |
|  |  |  |  |
| Pre-secondary | -0.34^***^ | -0.05 | -0.11 |
|  | (0.07) | (0.06) | (0.10) |
|  |  |  |  |
| Secondary | 0.02 | 0.19^**^ | -0.14 |
|  | (0.12) | (0.08) | (0.12) |
|  |  |  |  |
| Secondary Vocational | -0.13^*^ | 0.004 | -0.18^*^ |
|  | (0.08) | (0.06) | (0.09) |
|  |  |  |  |
| Tertiary Vocational | -0.20^**^ | 0.13^**^ | 0.04 |
|  | (0.10) | (0.06) | (0.09) |
|  |  |  |  |
| Constant | 0.30^***^ | 0.14^***^ | 0.19^**^ |
|  | (0.06) | (0.06) | (0.08) |
|  |  |  |  |
|  | | | |
| Observations | 1,308 | 3,593 | 1,276 |
| R^2^ | 0.06 | 0.08 | 0.12 |
| Residual Std. Error | 0.97 (df = 1300) | 0.96 (df = 3585) | 0.94 (df = 1268) |
| F Statistic | 12.26^***^ (df = 7; 1300) | 44.85^***^ (df = 7; 3585) | 25.35^***^ (df = 7; 1268) |
|  | | | |
| *Note:* Reference category for education is Tertiary Academic (University) ^*^p<0.1; ^**^p<0.05; ^***^p<0.01 | | | |

**Table S12.**

*Regression results of Benevolence scales on socio-demographics in the European Social Survey, the Longitudinal internet study for social sciences and the world value survey.*

|  | | | |
| --- | --- | --- | --- |
|  | ESS PVQ-21 | LISS RVS | WVS PVQ-11 |
|  | (1) | (2) | (3) |
|  | | | |
| age | 0.05^*^ | 0.08^***^ | 0.17^***^ |
|  | (0.03) | (0.02) | (0.03) |
|  |  |  |  |
| Male | -0.36^***^ | -0.25^***^ | -0.50^***^ |
|  | (0.05) | (0.03) | (0.05) |
|  |  |  |  |
| Primary | -0.03 | -0.20^**^ | 0.05 |
|  | (0.12) | (0.09) | (0.13) |
|  |  |  |  |
| Pre-secondary | 0.02 | -0.13^**^ | 0.10 |
|  | (0.07) | (0.06) | (0.10) |
|  |  |  |  |
| Secondary | 0.14 | 0.01 | 0.001 |
|  | (0.12) | (0.08) | (0.12) |
|  |  |  |  |
| Secondary Vocational | 0.12 | -0.05 | 0.05 |
|  | (0.08) | (0.06) | (0.09) |
|  |  |  |  |
| Tertiary Vocational | -0.01 | 0.02 | 0.15 |
|  | (0.10) | (0.06) | (0.09) |
|  |  |  |  |
| Constant | 0.14^**^ | 0.17^***^ | 0.15^*^ |
|  | (0.06) | (0.06) | (0.08) |
|  |  |  |  |
|  | | | |
| Observations | 1,308 | 3,594 | 1,272 |
| R^2^ | 0.04 | 0.02 | 0.10 |
| Adjusted R^2^ | 0.03 | 0.02 | 0.09 |
| Residual Std. Error | 0.98 (df = 1300) | 0.99 (df = 3586) | 0.95 (df = 1264) |
| F Statistic | 7.22^***^ (df = 7; 1300) | 12.10^***^ (df = 7; 3586) | 18.99^***^ (df = 7; 1264) |
|  | | | |
| *Note:* Reference category for education is Tertiary Academic (University) ^*^p<0.1; ^**^p<0.05; ^***^p<0.01 | | | |

**Table S13.**

*Regression results of Conformity scales on socio-demographics in the European Social Survey, the Longitudinal internet study for social sciences and the world value survey.*

|  | | | |
| --- | --- | --- | --- |
|  | *Dependent variable:* | | |
|  |  | | |
|  | ESS PVQ-21 | LISS RVS | WVS PVQ-11 |
|  | (1) | (2) | (3) |
|  | | | |
| age | 0.17^***^ | -0.02 | -0.04 |
|  | (0.03) | (0.02) | (0.03) |
|  |  |  |  |
| Male | 0.10^*^ | -0.02 | -0.03 |
|  | (0.05) | (0.03) | (0.06) |
|  |  |  |  |
| Primary | 0.48^***^ | 0.51^***^ | 0.29^**^ |
|  | (0.12) | (0.09) | (0.14) |
|  |  |  |  |
| Pre-secondary | 0.33^***^ | 0.60^***^ | 0.31^***^ |
|  | (0.07) | (0.06) | (0.10) |
|  |  |  |  |
| Secondary | 0.09 | 0.31^***^ | 0.26^**^ |
|  | (0.12) | (0.08) | (0.13) |
|  |  |  |  |
| Secondary Vocational | 0.26^***^ | 0.53^***^ | 0.37^***^ |
|  | (0.08) | (0.06) | (0.10) |
|  |  |  |  |
| Tertiary Vocational | 0.19^*^ | 0.14^**^ | 0.24^**^ |
|  | (0.10) | (0.06) | (0.10) |
|  |  |  |  |
| Constant | -0.25^***^ | -0.37^***^ | -0.25^***^ |
|  | (0.06) | (0.06) | (0.09) |
|  |  |  |  |
|  | | | |
| Observations | 1,308 | 3,594 | 1,260 |
| R^2^ | 0.07 | 0.05 | 0.01 |
| Adjusted R^2^ | 0.06 | 0.04 | 0.01 |
| Residual Std. Error | 0.97 (df = 1300) | 0.98 (df = 3586) | 1.00 (df = 1252) |
| F Statistic | 13.27^***^ (df = 7; 1300) | 24.85^***^ (df = 7; 3586) | 2.35^**^ (df = 7; 1252) |
|  | | | |

*Note:* Reference category for education is Tertiary Academic (University) ^*^p<0.1; ^**^p<0.05; ^***^p<0.01

**Table S14.**

*Regression results of Security scales on socio-demographics in the European Social Survey, the Longitudinal internet study for social sciences and the world value survey.*

|  | | | |
| --- | --- | --- | --- |
|  | *Dependent variable:* | | |
|  |  | | |
|  | ESS PVQ-21 | LISS RVS | WVS PVQ-11 |
|  | (1) | (2) | (3) |
|  | | | |
| age | 0.11^***^ | 0.12^***^ | 0.03 |
|  | (0.03) | (0.02) | (0.03) |
|  |  |  |  |
| Male | -0.21^***^ | -0.22^***^ | -0.11^**^ |
|  | (0.05) | (0.03) | (0.06) |
|  |  |  |  |
| Primary | 0.81^***^ | 0.44^***^ | 0.59^***^ |
|  | (0.12) | (0.09) | (0.14) |
|  |  |  |  |
| Pre-secondary | 0.62^***^ | 0.50^***^ | 0.59^***^ |
|  | (0.07) | (0.06) | (0.10) |
|  |  |  |  |
| Secondary | 0.44^***^ | 0.30^***^ | 0.47^***^ |
|  | (0.12) | (0.08) | (0.12) |
|  |  |  |  |
| Secondary Vocational | 0.42^***^ | 0.36^***^ | 0.47^***^ |
|  | (0.08) | (0.06) | (0.10) |
|  |  |  |  |
| Tertiary Vocational | 0.30^***^ | 0.29^***^ | 0.21^**^ |
|  | (0.10) | (0.06) | (0.09) |
|  |  |  |  |
| Constant | -0.27^***^ | -0.24^***^ | -0.32^***^ |
|  | (0.06) | (0.06) | (0.08) |
|  |  |  |  |
|  | | | |
| Observations | 1,308 | 3,593 | 1,271 |
| R^2^ | 0.11 | 0.05 | 0.05 |
| Adjusted R^2^ | 0.10 | 0.05 | 0.04 |
| Residual Std. Error | 0.95 (df = 1300) | 0.97 (df = 3585) | 0.98 (df = 1263) |
| F Statistic | 22.33^***^ (df = 7; 1300) | 28.53^***^ (df = 7; 3585) | 9.05^***^ (df = 7; 1263) |
|  | | | |

*Note:* Reference category for education is Tertiary Academic (University) ^*^p<0.1; ^**^p<0.05; ^***^p<0.01

**Table S15.**

*Regression results of Achievement scales on socio-demographics in the European Social Survey, the Longitudinal internet study for social sciences and the world value survey.*

|  | | | |
| --- | --- | --- | --- |
|  | *Dependent variable:* | | |
|  |  | | |
|  | ESS PVQ-21 | LISS RVS | WVS PVQ-11 |
|  | (1) | (2) | (3) |
|  | | | |
| age | -0.24^***^ | -0.06^***^ | -0.20^***^ |
|  | (0.03) | (0.02) | (0.03) |
|  |  |  |  |
| Male | 0.20^***^ | 0.31^***^ | 0.29^***^ |
|  | (0.05) | (0.03) | (0.05) |
|  |  |  |  |
| Primary | -0.51^***^ | -0.36^***^ | -0.45^***^ |
|  | (0.12) | (0.09) | (0.13) |
|  |  |  |  |
| Pre-secondary | -0.38^***^ | -0.50^***^ | -0.54^***^ |
|  | (0.07) | (0.06) | (0.10) |
|  |  |  |  |
| Secondary | -0.30^**^ | -0.54^***^ | -0.63^***^ |
|  | (0.12) | (0.08) | (0.12) |
|  |  |  |  |
| Secondary Vocational | -0.49^***^ | -0.50^***^ | -0.53^***^ |
|  | (0.08) | (0.06) | (0.09) |
|  |  |  |  |
| Tertiary Vocational | -0.24^**^ | -0.37^***^ | -0.43^***^ |
|  | (0.10) | (0.06) | (0.09) |
|  |  |  |  |
| Constant | 0.18^***^ | 0.27^***^ | 0.31^***^ |
|  | (0.05) | (0.06) | (0.08) |
|  |  |  |  |
|  | | | |
| Observations | 1,307 | 3,594 | 1,266 |
| R^2^ | 0.12 | 0.05 | 0.10 |
| Adjusted R^2^ | 0.11 | 0.05 | 0.10 |
| Residual Std. Error | 0.94 (df = 1299) | 0.97 (df = 3586) | 0.95 (df = 1258) |
| F Statistic | 24.49^***^ (df = 7; 1299) | 29.29^***^ (df = 7; 3586) | 20.66^***^ (df = 7; 1258) |
|  | | | |
| *Note:* Reference category for education is Tertiary Academic (University) ^*^p<0.1; ^**^p<0.05; ^***^p<0.01 | | | |

**Table S16.**

*Regression results of Hedonism scales on socio-demographics in the European Social Survey, the Longitudinal internet study for social sciences and the world value survey.*

|  | | | |
| --- | --- | --- | --- |
|  | *Dependent variable:* | | |
|  |  | | |
|  | ESS PVQ-21 | LISS RVS | WVS PVQ-11 |
|  | (1) | (2) | (3) |
|  | | | |
| age | -0.21^***^ | -0.25^***^ | -0.17^***^ |
|  | (0.03) | (0.02) | (0.03) |
|  |  |  |  |
| Male | -0.001 | 0.005 | 0.01 |
|  | (0.05) | (0.03) | (0.06) |
|  |  |  |  |
| Primary | 0.09 | 0.09 | -0.16 |
|  | (0.12) | (0.09) | (0.14) |
|  |  |  |  |
| Pre-secondary | 0.27^***^ | 0.05 | 0.15 |
|  | (0.07) | (0.06) | (0.10) |
|  |  |  |  |
| Secondary | 0.25^**^ | 0.16^**^ | 0.35^***^ |
|  | (0.12) | (0.08) | (0.13) |
|  |  |  |  |
| Secondary Vocational | 0.19^**^ | 0.08 | 0.09 |
|  | (0.08) | (0.06) | (0.10) |
|  |  |  |  |
| Tertiary Vocational | 0.02 | 0.12^*^ | 0.12 |
|  | (0.10) | (0.06) | (0.10) |
|  |  |  |  |
| Constant | -0.14^**^ | -0.08 | -0.11 |
|  | (0.06) | (0.06) | (0.08) |
|  |  |  |  |
|  | | | |
| Observations | 1,308 | 3,593 | 1,263 |
| R^2^ | 0.05 | 0.06 | 0.04 |
| Adjusted R^2^ | 0.04 | 0.06 | 0.03 |
| Residual Std. Error | 0.98 (df = 1300) | 0.97 (df = 3585) | 0.98 (df = 1255) |
| F Statistic | 9.43^***^ (df = 7; 1300) | 34.75^***^ (df = 7; 3585) | 7.49^***^ (df = 7; 1255) |
|  | | | |
| *Note:* Reference category for education is Tertiary Academic (University) ^*^p<0.1; ^**^p<0.05; ^***^p<0.01 | | | |

**Table S17.**

*Regression results of Stimulation on socio-demographics scales in the European Social Survey, the Longitudinal internet study for social sciences and the world value survey.*

|  | | | |
| --- | --- | --- | --- |
|  | *Dependent variable:* | | |
|  |  | | |
|  | ESS PVQ-21 | LISS RVS | WVS PVQ-11 |
|  | (1) | (2) | (3) |
|  | | | |
| age | -0.11^***^ | -0.15^***^ | -0.11^***^ |
|  | (0.03) | (0.02) | (0.03) |
|  |  |  |  |
| Male | 0.17^***^ | 0.36^***^ | 0.41^***^ |
|  | (0.05) | (0.03) | (0.06) |
|  |  |  |  |
| Primary | -0.54^***^ | -0.06 | -0.21 |
|  | (0.12) | (0.09) | (0.14) |
|  |  |  |  |
| Pre-secondary | -0.29^***^ | -0.04 | -0.19^*^ |
|  | (0.07) | (0.06) | (0.10) |
|  |  |  |  |
| Secondary | -0.37^***^ | -0.15^**^ | -0.16 |
|  | (0.12) | (0.08) | (0.12) |
|  |  |  |  |
| Secondary Vocational | -0.12 | -0.07 | -0.04 |
|  | (0.08) | (0.06) | (0.09) |
|  |  |  |  |
| Tertiary Vocational | 0.03 | -0.11^*^ | -0.12 |
|  | (0.10) | (0.06) | (0.09) |
|  |  |  |  |
| Constant | 0.08 | -0.09^*^ | -0.08 |
|  | (0.06) | (0.06) | (0.08) |
|  |  |  |  |
|  | | | |
| Observations | 1,308 | 3,593 | 1,261 |
| R^2^ | 0.05 | 0.06 | 0.06 |
| Adjusted R^2^ | 0.05 | 0.05 | 0.06 |
| Residual Std. Error | 0.97 (df = 1300) | 0.97 (df = 3585) | 0.97 (df = 1253) |
| F Statistic | 10.36^***^ (df = 7; 1300) | 30.11^***^ (df = 7; 3585) | 12.27^***^ (df = 7; 1253) |
|  | | | |
| *Note:* Reference category for education is Tertiary Academic (University) ^*^p<0.1; ^**^p<0.05; ^***^p<0.01 | | | |

**Table S18.**

*Regression results of Self-direction scales on socio-demographics in the European Social Survey, the Longitudinal internet study for social sciences and the world value survey.*

|  | | | |
| --- | --- | --- | --- |
|  | *Dependent variable:* | | |
|  |  | | |
|  | ESS PVQ-21 | LISS RVS | WVS PVQ-11 |
|  | (1) | (2) | (3) |
|  | | | |
| age | 0.03 | 0.09^***^ | -0.01 |
|  | (0.03) | (0.02) | (0.03) |
|  |  |  |  |
| Male | 0.07 | 0.10^***^ | 0.22^***^ |
|  | (0.05) | (0.03) | (0.06) |
|  |  |  |  |
| Primary | -0.55^***^ | -0.51^***^ | -0.48^***^ |
|  | (0.12) | (0.09) | (0.14) |
|  |  |  |  |
| Pre-secondary | -0.51^***^ | -0.57^***^ | -0.56^***^ |
|  | (0.07) | (0.06) | (0.10) |
|  |  |  |  |
| Secondary | -0.30^**^ | -0.27^***^ | -0.28^**^ |
|  | (0.12) | (0.08) | (0.13) |
|  |  |  |  |
| Secondary Vocational | -0.27^***^ | -0.43^***^ | -0.42^***^ |
|  | (0.08) | (0.06) | (0.10) |
|  |  |  |  |
| Tertiary Vocational | -0.09 | -0.24^***^ | -0.23^**^ |
|  | (0.10) | (0.06) | (0.10) |
|  |  |  |  |
| Constant | 0.23^***^ | 0.32^***^ | 0.24^***^ |
|  | (0.06) | (0.06) | (0.08) |
|  |  |  |  |
|  | | | |
| Observations | 1,308 | 3,594 | 1,248 |
| R^2^ | 0.05 | 0.04 | 0.05 |
| Adjusted R^2^ | 0.04 | 0.03 | 0.04 |
| Residual Std. Error | 0.98 (df = 1300) | 0.98 (df = 3586) | 0.98 (df = 1240) |
| F Statistic | 9.21^***^ (df = 7; 1300) | 18.73^***^ (df = 7; 3586) | 8.64^***^ (df = 7; 1240) |
|  | | | |
| *Note:* Reference category for education is Tertiary Academic (University) ^*^p<0.1; ^**^p<0.05; ^***^p<0.01 | | | |

**Table S19.**

Pairwise correlations between RVS ipsatized values scales with attitudinal questions on marriage, divorce, multicultural society and the big five personality traits.

| Variable | BE | UN | SD | ST | AC | CO | TR | HE | SE |
| --- | --- | --- | --- | --- | --- | --- | --- | --- | --- |
| Acculturate | -.15 | -.17 | -.16 | .04 | .03 | .16 | .04 | .05 | .09 |
| LR-scale | -.14 | -.22 | -.16 | .03 | .06 | .17 | .09 |  | .08 |
| Social desirable | .08 |  | -.09 | -.16 | -.08 | .21 | .14 | -.15 | .08 |
| ForGood | .13 | .17 | .14 |  | -.04 | -.16 |  | -.04 | -.09 |
| ForEasy | .08 | .13 | .14 |  |  | -.10 | -.07 | -.11 | -.06 |
| ForSS | .14 | .16 | .08 | -.05 | -.05 | -.12 | -.03 | -.03 |  |
| ForMany | -.16 | -.18 | -.19 | .05 |  | .16 | .03 | .07 | .11 |
| ForNeigh | -.09 | -.11 | -.11 |  |  | .10 | .04 | .07 | .05 |
| Extraversion | -.05 | -.05 | .05 | .16 | .05 | -.13 | -.07 | .05 | -.07 |
| Agreeableness | .22 | .22 | -.04 | -.13 | -.20 |  | .06 | -.09 | .06 |
| conscience |  | -.04 | -.09 | -.19 | -.07 | .36 | .12 | -.15 | .08 |
| emostab |  | -.03 | .03 | .04 |  | -.04 |  | .02 | -.04 |
| intellect | .08 | .04 | .26 |  |  | -.14 | -.03 | -.03 | -.13 |
| MarHappy |  | -.03 | .05 |  | .08 |  |  | -.09 |  |
| MarChild | -.04 | -.10 | -.03 | -.04 | .06 | .18 | .06 | -.18 | .06 |
| MarSingle |  | .03 |  |  |  |  |  | .04 |  |
| MarCoh1 |  | .07 |  |  | -.04 | -.16 | -.04 | .19 | -.06 |
| MarCoh2 | -.05 |  | .03 |  |  | -.09 | -.03 | .13 | -.03 |
| MarDiv1 | -.06 | .03 |  |  |  | -.04 |  | .07 | .06 |
| MarDiv2 | .03 | .08 |  |  | -.05 | -.16 | -.03 | .16 |  |

Note: Correlations are significant at p<0.05, insignificant correlations not shown. BE = Benevolence, UN = Universalism, SD = Self-direction, ST = Stimulation, AC = achievement, CO = conformity, TR = tradition, HE = Hedonism, SE = security.

Table S19 shows correlations between value scales, the big-five personality traits, attitudes on marriage, divorce, cohabitation (which should relate to Universalism, Benevolence, Conformity, Tradition), immigration and multicultural society (which should relate with Hedonism, Stimulation Self-direction, Conformity, Security). Overall the correlations show a statistically significant and expected negative or positive relationship, even though all are around the 0.15 in magnitude.

**Table S20.**

Item wordings and scales of questions used in Table S19.

| Item wording | Scale |
| --- | --- |
| ForGood: It is good if society consists of people from different cultures | 1 fully disagree  2 disagree  3 neither agree nor disagree  4 agree  5 fully agree |
| ForEasy: It should be made easier to obtain asylum in the Netherlands |  |
| ForSS: Legally residing foreigners should be entitled to the same social security as Dutch citizens |  |
| ForMany: There are too many people of foreign origin or descent in the Netherlands |  |
| ForNeigh: It does not help a neighborhood if many people of foreign origin or descent move in |  |
| Social Desirable (Marlowe-Crowne) | scored 1 point if respondents give a socially desirable answer. Sum of 10 questions. |
| Big Five Personality traits: extraversion, agreeableness, conscience, emostab: emotional stability, intellect | Personality was measured using 50 items from the International Personality Item Pool (IPIP). Total of 10 questions per trait, half reverse coded.  1 very inaccurate  2 moderately inaccurate  3 neither inaccurate nor accurate  4 moderately accurate  5 very accurate |
| Acculturate | Where would you place yourself on the scale 1-5:  1 immigrants can retain their own culture.  5 immigrants should adapt entirely to Dutch culture. |
| MarHappy: Married people are generally happier than unmarried people | 1 fully disagree  2 disagree  3 neither agree nor disagree  4 agree  5 fully agree |
| MarChild: People who want to have children should get married |  |
| MarSingle: A single parent can raise a child just as well as two parents together |  |
| MarCoh1: A couple may live together without intending to marry |  |
| MarCoh2: For couples who intend to marry, it is good to cohabit first. |  |
| MarDiv1: A divorce is the best option in case the couple is unable to resolve their marital issues. |  |
| MarDiv2: Young married couples with children are allowed to divorce |  |

The CFA models use item rating data from the 2013 wave of the LISS.To identify the CFA models, the first two factor loadings are fixed to 1 in the UN, AC, CO and STHE models. The STHE model includes two items for stimulation and one item for hedonism. Items with the best fit as identified in Table 4 were used.

**Table S20.**

**CFA Model Fit Indices**

| **Latent Variable** | **Chi-square** | **df** | **p-value** | **CFI** | **TLI** | **RMSEA** | **RMSEA CI Lower** | **RMSEA CI Upper** | **H0 RMSEA <= 0.05** | **SRMR** |
| --- | --- | --- | --- | --- | --- | --- | --- | --- | --- | --- |
| UN | 0.352 | 1 | 0.553 | 1.000 | 1.000 | 0.000 | 0.000 | 0.031 | 0.999 | 0.003 |
| BE | 178.395 | 6 | 0.000 | 0.983 | 0.972 | 0.075 | 0.066 | 0.085 | 0.000 | 0.024 |
| AC | 2.156 | 1 | 0.142 | 1.000 | 0.999 | 0.015 | 0.000 | 0.044 | 0.982 | 0.007 |
| CO | 2.527 | 1 | 0.112 | 1.000 | 0.999 | 0.017 | 0.000 | 0.045 | 0.976 | 0.009 |
| STHE | 0.341 | 1 | 0.559 | 1.000 | 1.001 | 0.000 | 0.000 | 0.031 | 0.999 | 0.003 |

Note. CFI = Comparative Fit Index; TLI = Tucker-Lewis Index; RMSEA = Root Mean Square Error of Approximation; SRMR = Standardized Root Mean Square Residual.
