# Supplementary material for: Constructing Schwartz values framework using the Rokeach values survey: Human value measurement in the longitudinal internet survey for social sciences
Source: PLoS One. 2025 Aug 12;20(8):e0329179. doi: 10.1371/journal.pone.0329179 (PMC12342246; doi:10.1371/journal.pone.0329179)
Supplement: S3 Code — SPSS, R and Stata Syntax for Computing Value Scales. (DOCX) [file pone.0329179.s003.docx]

## Stata syntax to create value scales.

/*

Data merging and ipsatization.

1. load wave 2008 of the LISS, merge it with relevant background files.

2. rename variables

3. check missing values

4. compute rating value scales

5. compute ipsatised value scales

*/

* system settings

set more off

version 17

clear

*paths for files

global dir "YOUR ROOT DIRECTORY"

global data "${dir}\Data Original\"

global dtaprc "${dir}\Data Processed\"

*--------------------------------------------------------------------------------

* 1. LOAD Data.

*--------------------------------------------------------------------------------

use "${data}cp08a_1p_EN.dta" ,clear

rename cp08a_m wave

duplicates report nomem_encr wave

*Background variables

merge 1:1 nomem_encr wave using "${data}\avars_200808_EN_2.0p.dta" , gen(back1)

duplicates report nomem_encr wave

drop if back1 ==2 // drop observations from background file that were not matched.

merge 1:1 nomem_encr wave using "${data}\avars_200805_EN_2.0p.dta" , gen(back2) update

duplicates report nomem_encr wave

drop if back2 ==2 // drop observations from background file that were not matched.

*--------------------------------------------------------------------------------

*2. make variable names shorter

* so that they do not have the three characters in the variable name which denote the wave e.g. the "08a" in cp08a001. If you rename variables in each wave, you can append them together. Be careful to check documentation that your variables have not changed names across waves!

*--------------------------------------------------------------------------------

foreach v of varlist cp* cv* {

local newname = subinstr("`v'", "08a","",.)

rename `v' `newname'

}

*--------------------------------------------------------------------------------

* 3. Check missing values

*--------------------------------------------------------------------------------

*Note: take out the following items if you do not want to include items with lower reliability: *Inner harmony (128), true friendship (126), Responsible (100) ,Pleasure (132), National security (124), Disciplined (109).*

misstable summarize cp099-cp134

*values should go from 1 to 7

* Rating items

global unitems " cp126 cp120 cp128 cp117 cp126 cp119"

global beitems "cp099 cp102 cp105 cp106 cp103 cp100"

global stitems "cp134 cp131"

global sditems "cp123 cp116"

global heitems "cp129"

global acitems "cp113 cp133 cp127"

global tritems “cp112 cp101”

global heitems “cp129 cp132”

global coitems " cp114 cp112 cp109 cp108"

*--------------------------------------------------------------------------------

* 4. Ratings

*--------------------------------------------------------------------------------

/*NOTE: help egen states that rowmean(): creates the (row) means of the variables in varlist, ignoring missing values. For example, if three variables are specified and, in some observations, one of the variables is missing, in those observations newvar will contain the mean of the two variables that do exist. Other observations will contain the mean of all three variables. If all values in varlist are missing for an observation, newvar is set to missing for that observation.

*/

egen coitems = rowmean($coitems)

egen unitems = rowmean($unitems)

egen beitems = rowmean($beitems)

egen sditems = rowmean($sditems)

egen stitems = rowmean($stitems)

egen heitems = rowmean($heitems)

egen acitems = rowmean($acitems)

egen tritems = rowmean($tritems)

egen seitems = rowmean(cp124)

*get mean rating weighted by the number of items per value

egen mrat = rowmean(coitems unitems beitems sditems stitems heitems acitems tritems seitems)

*label variables

lab var coitems "Conformity rating"

lab var unitems "Universalism rating"

lab var beitems "Benevolance rating"

lab var sditems "Self-direction rating"

lab var stitems "Stimulation rating"

lab var heitems "Hedonism rating"

lab var acitems "Achievement rating"

lab var tritems “Tradition rating”

lab var seitems “Secutity rating”

*--------------------------------------------------------------------------------

* 5. Create Ipsatised value measures

*--------------------------------------------------------------------------------

foreach v of varlist coitems unitems beitems sditems stitems heitems acitems tritems seitems {

*generate ipsatised value

gen ip_`v' = `v' -mrat

*create new variable label

local oldlab: variable label `v'

local newlab = subinstr("`oldlab'","rating", "ipsatised" ,1)

lab var ip_`v' "`newlab'"

}

* SAVE

compress

save "${dtaprc}Wave2008.dta" ,replace

## SPSS syntax to create value scales.

* Encoding: UTF-8.

/* Data merging and ipsatization.

*1. load wave 2008 of the liss, merge it with relevant background files.

*2. rename variables

*3. check missing values

*4. compute rating value scales

*5. compute ipsatised value scales

*--------------------------------------------------------------------------------

* 1. LOAD Data.

*--------------------------------------------------------------------------------

GET

FILE='your root directory/cp08a_1p_EN.sav'.

*********************************************************************************

** rename variable to wave to prevent double variable in combined dataset**

**********************************************************************************

RENAME VARIABLES cp08a_m = wave.

EXECUTE.

**********************************************************************************

** get the background variables for the respondents from the same time period*

**********************************************************************************

GET FILE=''your root directory/avars_200808_EN_2.0p.sav'.

**********************************************************************************

**combine the datasets**

**********************************************************************************

SORT CASES BY nomem_encr nohouse_encr.

DATASET ACTIVATE DataSet4.

SORT CASES BY nomem_encr nohouse_encr.

DATASET ACTIVATE DataSet1.

MATCH FILES /FILE=*

/FILE='DataSet4'

/BY nomem_encr nohouse_encr.

EXECUTE.

**********************************************************************************

**filter out respondents without content in 2008 personality

**********************************************************************************

FILTER OFF.

USE ALL.

SELECT IF (nmiss (cp08a_m) = 0).

EXECUTE.

**********************************************************************************

***shorten the variables****

**********************************************************************************

RENAME VARIABLES

cp08a001 = cp001

cp08a002 = cp002

cp08a003 = cp003

cp08a004 = cp004

cp08a005 = cp005

cp08a006 = cp006

cp08a007 = cp007

cp08a008 = cp008

cp08a009 = cp009

cp08a010 = cp010

cp08a011 = cp011

cp08a012 = cp012

cp08a013 = cp013

cp08a014 = cp014

cp08a015 = cp015

cp08a016 = cp016

cp08a017 = cp017

cp08a018 = cp018

cp08a019 = cp019

cp08a020 = cp020

cp08a021 = cp021

cp08a022 = cp022

cp08a023 = cp023

cp08a024 = cp024

cp08a025 = cp025

cp08a026 = cp026

cp08a027 = cp027

cp08a028 = cp028

cp08a029 = cp029

cp08a030 = cp030

cp08a031 = cp031

cp08a032 = cp032

cp08a033 = cp033

cp08a034 = cp034

cp08a035 = cp035

cp08a036 = cp036

cp08a037 = cp037

cp08a038 = cp038

cp08a039 = cp039

cp08a040 = cp040

cp08a041 = cp041

cp08a042 = cp042

cp08a043 = cp043

cp08a044 = cp044

cp08a045 = cp045

cp08a046 = cp046

cp08a047 = cp047

cp08a048 = cp048

cp08a049 = cp049

cp08a050 = cp050

cp08a051 = cp051

cp08a052 = cp052

cp08a053 = cp053

cp08a054 = cp054

cp08a055 = cp055

cp08a056 = cp056

cp08a057 = cp057

cp08a058 = cp058

cp08a059 = cp059

cp08a060 = cp060

cp08a061 = cp061

cp08a062 = cp062

cp08a063 = cp063

cp08a064 = cp064

cp08a065 = cp065

cp08a066 = cp066

cp08a067 = cp067

cp08a068 = cp068

cp08a069 = cp069

cp08a070 = cp070

cp08a071 = cp071

cp08a072 = cp072

cp08a073 = cp073

cp08a074 = cp074

cp08a075 = cp075

cp08a076 = cp076

cp08a077 = cp077

cp08a078 = cp078

cp08a079 = cp079

cp08a080 = cp080

cp08a081 = cp081

cp08a082 = cp082

cp08a083 = cp083

cp08a084 = cp084

cp08a085 = cp085

cp08a086 = cp086

cp08a087 = cp087

cp08a088 = cp088

cp08a089 = cp089

cp08a090 = cp090

cp08a091 = cp091

cp08a092 = cp092

cp08a093 = cp093

cp08a094 = cp094

cp08a095 = cp095

cp08a096 = cp096

cp08a097 = cp097

cp08a098 = cp098

cp08a099 = cp099

cp08a100 = cp100

cp08a101 = cp101

cp08a102 = cp102

cp08a103 = cp103

cp08a104 = cp104

cp08a105 = cp105

cp08a106 = cp106

cp08a107 = cp107

cp08a108 = cp108

cp08a109 = cp109

cp08a110 = cp110

cp08a111 = cp111

cp08a112 = cp112

cp08a113 = cp113

cp08a114 = cp114

cp08a115 = cp115

cp08a116 = cp116

cp08a117 = cp117

cp08a118 = cp118

cp08a119 = cp119

cp08a120 = cp120

cp08a121 = cp121

cp08a122 = cp122

cp08a123 = cp123

cp08a124 = cp124

cp08a125 = cp125

cp08a126 = cp126

cp08a127 = cp127

cp08a128 = cp128

cp08a129 = cp129

cp08a130 = cp130

cp08a131 = cp131

cp08a132 = cp132

cp08a133 = cp133

cp08a134 = cp134

cp08a135 = cp135

cp08a136 = cp136

cp08a137 = cp137

cp08a138 = cp138

cp08a139 = cp139

cp08a140 = cp140

cp08a141 = cp141

cp08a142 = cp142

cp08a143 = cp143

cp08a144 = cp144

cp08a145 = cp145

cp08a146 = cp146

cp08a147 = cp147

cp08a148 = cp148

cp08a149 = cp149

cp08a150 = cp150

cp08a151 = cp151

cp08a152 = cp152

cp08a153 = cp153

cp08a154 = cp154

cp08a155 = cp155

cp08a156 = cp156

cp08a157 = cp157

cp08a158 = cp158

cp08a159 = cp159

cp08a160 = cp160

cp08a161 = cp161

cp08a162 = cp162

cp08a163 = cp163

cp08a164 = cp164

cp08a165 = cp165

cp08a166 = cp166

cp08a167 = cp167

cp08a168 = cp168

cp08a169 = cp169

cp08a170 = cp170

cp08a171 = cp171

cp08a172 = cp172

cp08a173 = cp173

cp08a174 = cp174

cp08a175 = cp175

cp08a176 = cp176

cp08a177 = cp177

cp08a178 = cp178

cp08a179 = cp179

cp08a180 = cp180

cp08a181 = cp181

cp08a182 = cp182

cp08a183 = cp183

cp08a184 = cp184

cp08a185 = cp185

cp08a186 = cp186

cp08a187 = cp187

cp08a188 = cp188

cp08a189 = cp189

cp08a190 = cp190

cp08a191 = cp191

cp08a192 = cp192

cp08a193 = cp193.

execute.

*********************************************************************************** 3. Check missing values

**********************************************************************************

* Define missing values for variables cp099 to cp134. (anything above 7)

MISSING VALUES cp099 to cp134 (8 THRU HI).

* Summarize variables.

FREQUENCIES VARIABLES=cp099 TO cp134

/ORDER=ANALYSIS.

**********************************************************************************

**** compute the values****

**********************************************************************************

*Calculate the mean for raw value scores.

COMPUTE coitems = MEAN(cp108, cp112, cp114).

COMPUTE unitems = MEAN(cp117, cp120, cp119).

COMPUTE beitems = MEAN(cp102, cp103, cp099, cp106, cp105).

COMPUTE sditems = MEAN(cp123, cp116).

COMPUTE stitems = MEAN(cp134, cp131).

COMPUTE heitems = MEAN(cp129).

COMPUTE acitems = MEAN(cp113, cp133, cp127).

EXECUTE.

** add variable labels

*label variables

Variable labels

coitems 'Conformity rating'

unitems 'Universalism rating'

beitems 'Benevolence rating'

sditems 'Self-direction rating'

stitems 'Stimulation rating'

heitems 'Hedonism rating'

acitems 'Achievement rating'.

EXECUTE.

**********************************************************************************

*show the descriptives of the raw values for visual check

**********************************************************************************

FREQUENCIES VARIABLES=coitems unitems beitems sditems stitems heitems acitems

/STATISTICS=RANGE MINIMUM MAXIMUM STDDEV MEAN MEDIAN

/FORMAT=NOTABLE

/ORDER=ANALYSIS.

EXECUTE.

*********************************************************************************

*compute the MRAT score for the ipsatization of the values

**********************************************************************************

Compute mrat = mean (heitems, coitems, unitems, beitems, sditems, stitems, acitems).

EXECUTE.

**********************************************************************************

*Calculate the mean for raw value scores.

**********************************************************************************COMPUTE coitems_ips =coitems - mrat.

COMPUTE unitems_ips =unitems- mrat.

COMPUTE beitems_ips = beitems- mrat.

COMPUTE sditems_ips =sditems- mrat.

COMPUTE stitems_ips = stitems- mrat.

COMPUTE heitems_ips = heitems- mrat.

COMPUTE acitems_ips = acitems- mrat.

EXECUTE.

**********************************************************************************

* show the descriptives of the ipsatized values

**********************************************************************************

FREQUENCIES VARIABLES=coitems_ips unitems_ips beitems_ips sditems_ips stitems_ips heitems_ips acitems_ips

/STATISTICS=RANGE MINIMUM MAXIMUM STDDEV MEAN MEDIAN

/FORMAT=NOTABLE

/ORDER=ANALYSIS.

**********************************************************************************

* save your file with new name

**********************************************************************************

SAVE OUTFILE=

'your root directory/Wave2008.sav'

/COMPRESSED.
